# Assessing the protective effects of different surface coatings on NaYF_4_:Yb^3+^, Er^3+^ upconverting nanoparticles in buffer and DMEM

**DOI:** 10.1038/s41598-020-76116-z

**Published:** 2020-11-09

**Authors:** Maysoon I. Saleh, Bastian Rühle, Shu Wang, Jörg Radnik, Yi You, Ute Resch-Genger

**Affiliations:** 1 Federal Institute for Materials Research and Testing, Division 1.2 Biophotonics, Richard-Willstätter-Str. 11, 12489 Berlin, Germany; 2 Institut Für Chemie Und Biochemie, Freie Universität Berlin, Takustrasse 3, 14195 Berlin, Germany; 3 Federal Institute for Materials Research and Testing, Division 6.1, Unter den Eichen 44-46, 12203 Berlin, Germany; 4 Federal Institute for Materials Research and Testing, Division 6.3, structural analysis, Richard-Willstätter-Str. 11, 12489 Berlin, Germany

**Keywords:** Chemistry, Materials science, Nanoscience and technology

## Abstract

We studied the dissolution behavior of β NaYF_4_:Yb(20%), Er(2%) UCNP of two different sizes in biologically relevant media i.e., water (neutral pH), phosphate buffered saline (PBS), and Dulbecco’s modified Eagle medium (DMEM) at different temperatures and particle concentrations. Special emphasis was dedicated to assess the influence of different surface functionalizations, particularly the potential of mesoporous and microporous silica shells of different thicknesses for UCNP stabilization and protection. Dissolution was quantified electrochemically using a fluoride ion selective electrode (ISE) and by inductively coupled plasma optical emission spectrometry (ICP OES). In addition, dissolution was monitored fluorometrically. These experiments revealed that a thick microporous silica shell drastically decreased dissolution. Our results also underline the critical influence of the chemical composition of the aqueous environment on UCNP dissolution. In DMEM, we observed the formation of a layer of adsorbed molecules on the UCNP surface that protected the UCNP from dissolution and enhanced their fluorescence. Examination of this layer by X-ray photoelectron spectroscopy (XPS) and mass spectrometry (MS) suggested that mainly phenylalanine, lysine, and glucose are adsorbed from DMEM. These findings should be considered in the future for cellular toxicity studies with UCNP and other nanoparticles and the design of new biocompatible surface coatings.

## Introduction

Lanthanide-doped upconversion nanoparticles (UCNP) such as NaYF_4_:Yb,Er and NaYF_4_:Yb,Tm have been in the focus of many studies in the last two decades due to their unique optical properties. This includes multiphotonic excitation in the near-infrared (NIR) region^1–6^, emission of a multitude of characteristic narrow emission bands at shorter (upconversion luminescence, UCL) and longer (downshifted luminescence, DSL) wavelengths than the exciting photons^7^, long luminescence lifetimes^8^, and a high photostability^7^. These properties make them attractive fluorescent reporters for life sciences applications such as bioimaging^9,10^, in-vitro and in-vivo detection of biomolecules^11–14^, drug delivery^15^, photodynamic therapy^2,9,10,16^, and biosensing^2,3^. Utilization in bioimaging and cellular studies requires, however, biocompatible particles that are sufficiently stable in diluted dispersions and have no acute or chronic toxicity under application-relevant conditions^17–21^. In the case of UCNP, this implies they should not release potentially toxic constituents such as fluoride and lanthanide ions.

UCNP are commonly synthesized in apolar solvents using hydrophobic oleic acid as a capping agent^2,22,23^, which makes them dispersible only in apolar solvents. To render them water dispersible for applications in biological systems, they must undergo surface modification either by ligand exchange or addition of a new coating (ligand addition or encapsulation) on top of the initially present surface ligands^24–26^. Several ligand exchange procedures have been reported so far^24,25,27–31^, such as the exchange of the oleate ligands for polyacrylic acid (PAA)^6^, polyethylene glycol (PEG)^7^, phosphonate ligands^32^, citrate^8^, or removal of the oleate ligand with NOBF_4_/DMF yielding “naked” UCNP stabilized with electrostatically adsorbed BF_4_^¯^^9^. Alternatively, silica coatings have been used that also enable further surface functionalization^33^, such as the covalent attachment of targeted biomolecules and sensor molecules^2,10^. UCNP surface chemistry is of considerable importance not only for shielding the lanthanide ions of the UCNP from luminescence quenching molecules that contain moieties with high energy vibrations such as –OH^34–36^, but also for UCNP dispersibility and colloidal stability in aqueous environments and the prevention of UCNP dissolution upon dilution, thereby minimizing toxicity concerns^21,37^. For years, UCNP were assumed to be chemically inert, due to the very low solubility products of lanthanide fluorides in water (e. g., K_sp_ = 3.26 × 10^–21^ for LaF_3_)^38^. The influence of size, surface-to-volume ratio, and surface curvature on solubility products that are usually determined for larger size powders or bulk materials was neglected. However, there appeared an increasing number of reports recently on the dissolution of UCNP like NaYF_4_ -based materials in aqueous environments under high dilution conditions ^14–18^, leading to the release of fluoride and lanthanide ions^30,39–43^. This raised concerns of UCNP biocompatibility and triggered toxicity studies^17^.

In this work, we studied the stability of 20 nm and 30 nm-sized *β*-NaYF_4_:Yb(20%), Er(2%) UCNP from one batch each with different surface chemistries such as polyacrylic acid (PAA) and citrate ligands as well as mesoporous and microporous silica shells in different aqueous environments. Parameters addressed besides size and surface chemistry include UCNP concentration (5 mg/L, 50 mg/L), temperature (room temperature and body temperature), and the chemical composition of typically used aqueous environments like water (neutral pH), PBS, and cell culture medium (DMEM). The release of fluoride and lanthanide ions was quantified with a fluoride ion-selective electrode (ISE) and by inductively coupled plasma optical emission spectrometry (ICP-OES), respectively. Size- and environment-sensitive upconversion luminescence (UCL) spectra and luminescence lifetimes were utilized for UCNP stability monitoring. The investigation of organic molecules adsorbed on the surface of UCNP aged in DMEM was conducted by X-ray photoelectron spectroscopy (XPS) and mass spectrometry (MS).

## Experimental

### Materials

Lanthanide chlorides with high purity (99.99%) were used for the synthesis of the UCNP. Sodium oleate (82%), oleic acid (90%, technical grade), octadecene (90%, technical grade), hexadecyltrimethylammonium bromide (CTAB, 98%), citric acid (99.5%), diethylene glycol (DEG, 99%), N,N-dimethyl formamide (DMF, 99.5%), nitrosyl tetrafluoroborate (95%), polyacrylic acid (MW = 1800 Da), trisodium citrate dihydrate (95%), tetraethyl orthosilicate for synthesis (98%), and ammonia solution (25% wt% in water) were obtained from Sigma Aldrich (Germany) and used without further purification. ICP standard solutions (1000 mg/L in nitric acid) used for calibration of the measurements quantifying fluoride and lanthanide ions were purchased from Sigma Aldrich. Dulbecco’s Modified Eagle Medium (DMEM) (product number: D5546) was purchased from Sigma Aldrich. The detailed composition of DMEM can be found in the supplementary information (Table S2, SI). All solvents employed for the optical measurements were purchased from Sigma Aldrich in spectroscopic grade. Water refers here to Milli-Q water.

### Synthesis and surface modifications of the UCNP

NaYF_4_:20%Yb^3+^,2%Er^3+^ UCNP were synthesized according to a previously reported procedure^26–28^ with some modifications^25,44^. Surface modifications and silica coating experiments followed published procedures^45–49^. The detailed synthesis and characterization of the UCNP are provided in the supplementary information (SI).

Surface modified UCNP were stored in absolute ethanol, except for bare UCNP that were stored in dimethyl formamide (DMF). Stock solutions of the different UCNP (5 mg/mL) were stored in the fridge (at 4 °C) after surface modification and prior to their use in aging studies.

### Structural analysis

#### Electron microscopy

Transmission scanning electron microscopy (TSEM) images were recorded with a Hitachi SU 8030 scanning electron microscope in TSEM mode with an electron acceleration voltage of 30 kV and a current of 20 μA. A droplet of a dilute dispersion of the particles was dried on a carbon-coated copper grid (Cu 400 mesh, Quantifoil).

#### Zeta potential measurements

Zeta potential measurements of the surface-modified UCNP were performed with a DLS Zeta Potential Analyzer, Zetasizer Nano ZS90 (Malvern). Measurements were carried out for UCNP dispersions in MilliQ water at room temperature, the samples were placed in Zetasizer Nano ZS in capillary zeta cells DTS 1070 from Malvern Instruments.

#### X-ray diffraction measurements

X-ray diffraction measurements were performed with a STOE STADI P powder diffractometer with a Cu tube, a scan speed of 0.2-degree step/120 s, a tube voltage of 40 kV and a tube current of 30 mA in transmission geometry.

#### Absorption and fluorescence measurements

Absorption spectra were measured with a CARY 5000 absorption spectrometer (Varian) and fluorescence spectra were obtained with a calibrated FSP920 fluorescence spectrometer (Edinburgh Instruments) equipped with a xenon lamp and a 2 W 980 nm laser diode. Emission decay kinetics and lifetimes were obtained with FLS980 and FLS920 fluorescence lifetime spectrometers (Edinburgh Instruments). All measurements were carried out at room temperature in 1 cm quartz cells (Hellma) using an excitation wavelength of 976 nm and identical measurement conditions (i.e., excitation power density etc.). The lifetimes of the Yb^3+^ and Er^3+^ emission bands were obtained from the decay profiles recorded at 1010 nm and 545 nm using a tail fit with a biexponential decay. For the time-resolved UCL studies, all samples were dispersed in water and the measurements were carried out at the same excitation power density (75 W/cm^2^) to enable a direct comparison of the excitation power density-dependent UCL behavior.

#### Quantification of the components released from the UCNP

Quantification of the released lanthanide ions was carried out by ICP-OES using a Model: FHX, 76004553 spectrometer from SPECTRO Arcos-EOP. The amount of released fluoride ions was determined with a fluoride ion-selective electrode (ISE) from Metrohm AG, Switzerland.

#### XPS measurements

The XPS spectra of UCNP were measured with an AXIS Ultra DLD photoelectron spectrometer (Kratos Analytical, Manchester, UK) with monochromatic Al Kα radiation (E = 1486.6 eV) at a pressure below 1 × 10^–8^ mbar. The electron emission angle was 0° and the source-to-analyzer angle was 60°. The binding energy scale of the instrument was calibrated following a procedure from Kratos which uses ISO 15472 binding energy data^50^. XPS spectra were recorded by setting the instrument to the hybrid lens mode and the slot mode with a 300 × 700 µm^2^ analysis area. Furthermore, the charge neutralizer was used. All spectra were recorded in the fixed analyzer transmission (FAT) mode. The binding energies were referenced to the C1s peak of aliphatic carbon at 285.0 eV. Before determining the peak area and fitting, the background was subtracted using a modified Toogaard background^51^. For the quantification, the survey spectra obtained with a pass energy of 80 eV were used. The peak intensities were corrected for the appropriate Scofield factors, the inelastic mean free path, and the intensity response function of the instrument. The maximum relative uncertainty for the composition was ± 20%. The chemical species were determined with fits of the peaks measured at high resolution (pass energy of 20 eV). For the fits, a combination of Gaussian–Lorentzian peak profiles was used^51^. The uncertainty of the binding energy was around ± 0.4 eV. For sample preparation, 1 mg of UCNP-Bare-20 nm was redispersed in 1 mL of DMEM for one hour at room temperature under vortex shaking, the sample was centrifuged, and the supernatant was removed. The pellet was redispersed in 10µL of water and the solution was drop-casted on a silicon wafer (cut in about 1 × 1 cm^2^ shaped pieces).

#### MS measurements

A unit-resolution 3D ion trap mass spectrometer (Thermo LCQ Deca XP) was used to record all mass spectra. The mass range was set to *m/z* 50 to 450. The automatic gain control (AGC) was disabled to maintain a consistent mass-spectral acquisition rate at ~ 3 spectra/s. The injection time was set to 45 ms at 1 micro scan (without hardware averaging). The ionization method used has been previously described^52^. An in-house built device similar to a surface acoustic wave nebulizer (SAWN) was used to introduce the sample in a pulsed fashion. The sample was first mixed with methanol (HPLC grade) in a volumetric ratio of 1:2. In each sample analysis, 10 µL of the sample solution was applied on the surface of the nebulizer. The nebulizer was activated for 10 s each time. This pulsed sample introduction allowed the application of cross correlation for data processing^53^. Analyte-related ions appeared only during this period, because the samples were introduced in a defined time window. The background ions were automatically flagged and removed from the mass spectra.

For sample preparation, 1 mg of UCNP-bare-20 was incubated in 1 mL of DMEM for 1 h at room temperature under vortex shaking (same conditions as for XPS). The sample was centrifuged, the supernatant was removed, redispersed in 100µL of Phosphate Buffer (100 mM; pH 7.4) and incubated for 6 h at 60 °C to dissolve the particles and release the adsorbed molecules from the particle surface. Afterwards, the sample was centrifuged for 15 min at 16.1 k rcf to remove insoluble components, and the supernatant was analyzed by MS.

### Aging experiments

**Figure 1:**
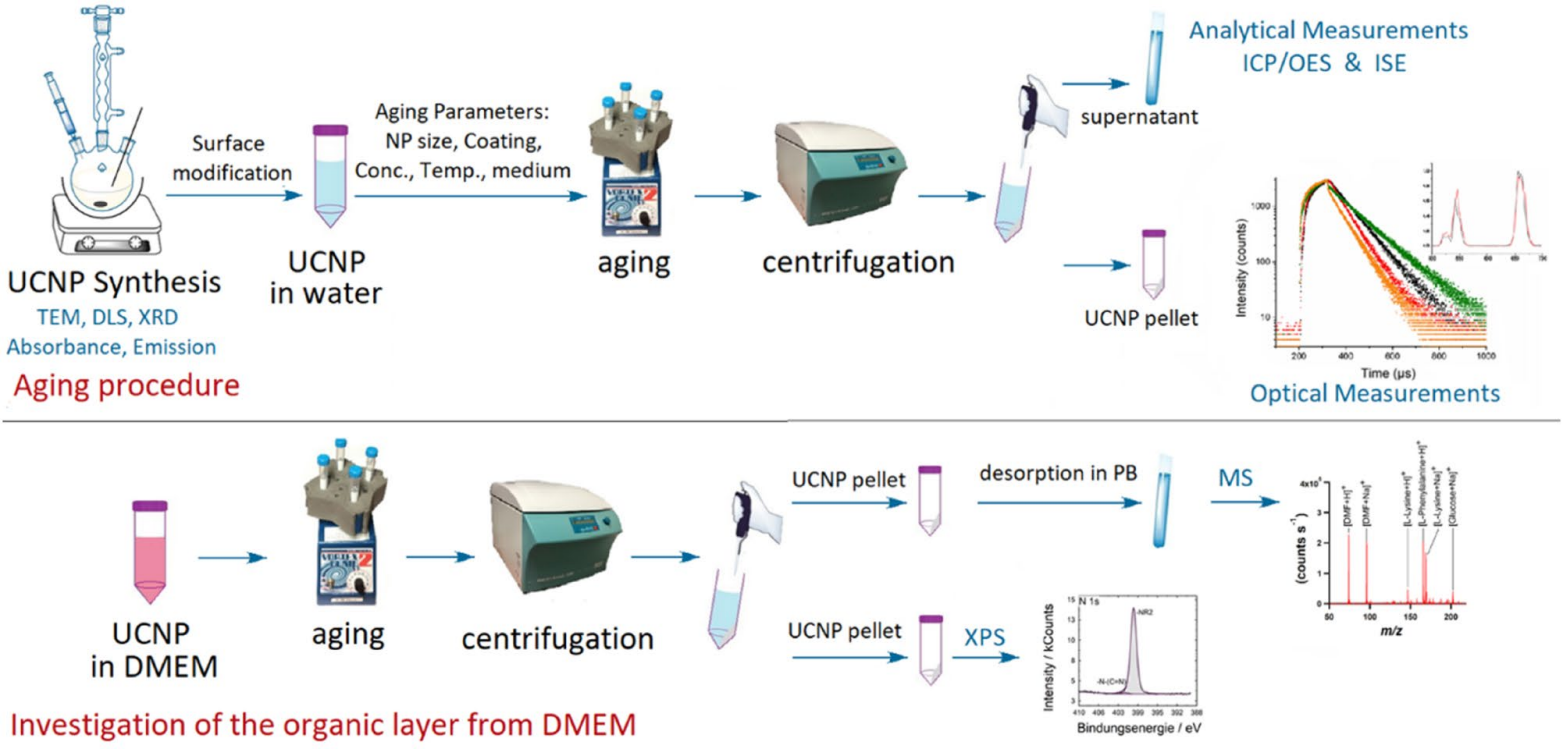
Schematic representation of the aging procedure used here for the different UCNP, exemplarily shown for water.

The desired amount of UCNP (0.005 mg for starting concentration of 5 mg/L or 0.05 mg for starting concentration 50 mg/L) was re-dispersed in 10 mL of the aqueous medium of interest in a 15 mL centrifuge tube, and then kept on a vortex shaker for six hours. Afterwards, the solution was centrifuged, the supernatant was collected with a pipette, the residual UCNP were re-dispersed in 10 mL of the same aqueous medium and allowed to shake on the vortex shaker for another nine hours. The supernatant was collected again, kept after each centrifugation step for ICP-OES and ISE measurements, and the same procedure was repeated for all other time points of the experiments. Additionally, the solid UCNP residue after the last time point was used for optical measurements. A schematic representation of the procedure is shown in Fig. 1.

## Results and discussion

The samples studied and abbreviations used, reflecting their core size and surface chemistry, are summarized in Table 1. As summarized in Fig. 1, the as-synthesized UCNP and the surface modified UCNP were characterized by transmission scanning electron microscopy (TSEM) images, absorption and emission spectra as well as X-ray diffraction (XRD) diffractograms that are shown in the SI (Figures S1-S4). Thin (10 nm) and a thick (73 nm) microporous silica shells as well as a mesoporous silica shell (23 nm) were applied to 30 nm UCNP, and the corresponding TSEM images are shown in the SI (Figure S1). Ligand exchange was supported by zeta potential measurements as shown in the supplementary information (Table S3, SI). 20 nm and 30 nm-sized β-NaYF_4_:20%Yb^3+^, 2%Er^3+^ UCNP with different surface chemistries were dispersed in concentrations of 5 and 50 mg/L in water, PBS, and DMEM and aged for time intervals of 6, 15, 24, 48, and 72 h at room temperature and at 37 °C. The supernatants were collected from the samples after aging and were kept for further analysis of the released ions by ICP-OES and ISE measurements.

**Table 1:** UCNP samples used for the aging studies including terminology used.

UCNP with different surface coatings		
Sample ID	Surface coating	Diameter (nm)
UC-bare-20*	BF_4_^¯^	20
UC-bare-30*	BF_4_^¯^	30
UC-citrate-20	Citrate	20
UC-citrate-30	Citrate	30
UC-PAA-20	Polyacrylic acid	20
UC-PAA-30	Polyacrylic acid	30

UCNP coated with silica (UCNP diameter for all samples is 30 nm)		
Sample ID	Shell porosity	Shell thickness (nm)
UC-mSiO_2_-NH_3_	Mesoporous	23
UC-mSiO_2_-NaOH	Mesoporous	23
UC-SiO_2_-thin	Microporous	10
UC-SiO_2_-thick	Microporous	73

*Oleate ligands were removed and the UCNP were electrostatically stabilized by surface adsorbed BF_4_^¯^ after ligand exchange.

As the aging experiments were performed for UCNP of different core sizes, surface coatings, and starting concentrations, a direct comparison of the results based solely on the moles of released ions obtained from ICP-OES and ISE measurements can be misleading. To consider UCNP size and concentration, we therefore calculated the fractions of the number of released ions of the element of interest to the total number of ions of the same element present in the UCNP before aging for quantifying ion release and comparing the different UCNP types and aging scenarios. In the following, this quantity is referred to as “mole fraction of the element” for convenience, bearing in mind that this definition deviates from the conventional definition of a mole fraction of an element in a given chemical system.

### Influence of UCNP size and surface ligand on dissolution

**Figure 2:**
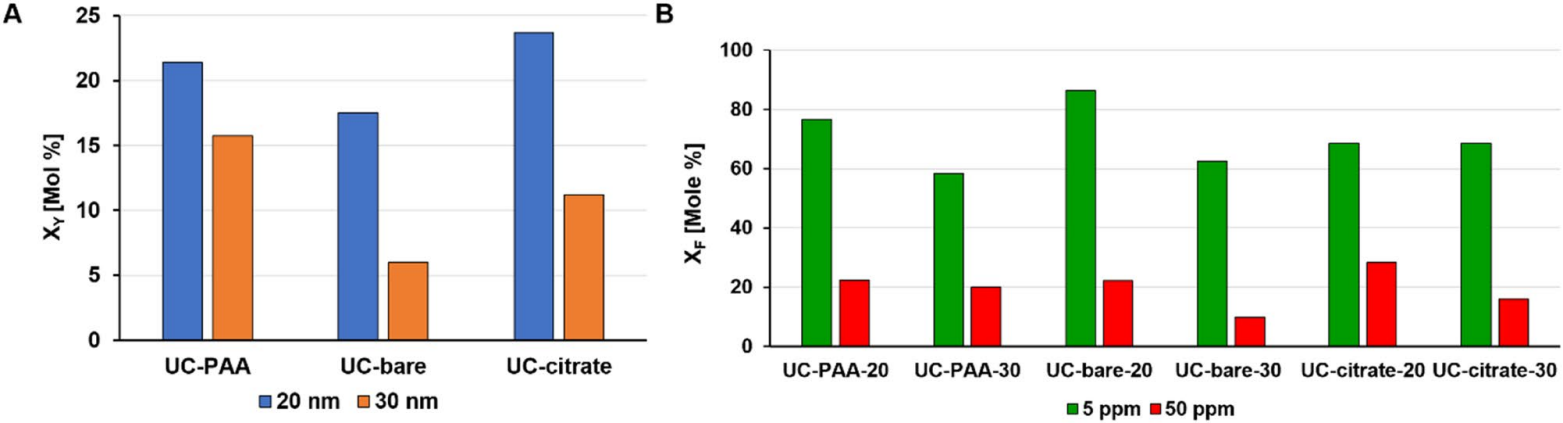
(**A)** Mole fractions of Y^3+^ released from UCNP with different surface ligands determined for two core sizes (blue: 20 nm, orange: 30 nm) after 72 h of aging in water at room temperature. (**B)** Mole fractions of F^−^ released from UCNP of different particle size and surface functionalizations determined for two different initial UCNP concentrations (green: 5 mg/L, red: 50 mg/L). The samples were aged in water at room temperature for 72 h.

The results of the stability studies with differently sized UCNP bearing either coordinatively bound surface ligands or with ligand-free (bare) UCNP are summarized in Fig. 2. As shown in Fig. 2A, ion release was observed for all UCNP regardless of their surface chemistry. Moreover, the relative percentages of the released ions were consistent with the composition of the unaged UCNP (78% Y^3+^, 20% Yb^3+^ and 2% Er^3+^), see Table S4, SI. Therefore, only the results of Y^3+^ and F^-^ are presented in the following sections. The Y^3+^ ion was chosen because it is the main constituent of the UCNP and hence its detection in the supernatant by ICP-OES can be achieved with a higher accuracy and a smaller uncertainty than the quantification of less abundant Yb^3+^ and Er^3+^.

The fractions of the released ions clearly depended on UCNP concentration and chemical nature of the surface coatings (Figure S5, SI). 20 nm UCNP (blue bars) have a higher tendency to release lanthanide ions and dissolve than their 30 nm UCNP counterparts (orange bars) with a similar surface chemistry. The same trend was observed for the release of fluoride ions (Fig. 2B). This highlights the influence of both the surface area in contact with the surrounding medium and nanoparticle curvature. However, the influence of UCNP surface chemistry on particle dissolution was small compared to the influence of the chemical composition of the aqueous medium used for the aging studies.

### Effect of UCNP concentration

For bare and ligand stabilized UCNP, ion release in water was more significant for diluted UCNP dispersions with particle concentrations of 5 mg/L compared to UCNP dispersions containing 50 mg/L of nanoparticles. This trend was observed for all UCNP dispersions studied regardless of UCNP surface coating (Fig. 2B). This observation reflects the trend reported e.g., by Dukhno et al.^54^, and is in agreement with observations with other types of nanoparticles like semiconductor quantum dots with coordinatively bound surface ligands that can desorb upon dilution. In principle, UCNP dissolution can be prevented by addition of NaF, as shown by other groups^54,55^, which shifts the dissolution equilibrium towards the solid, i.e., nanoparticle system. However, adding potentially hazardous fluoride ions is not an option for applying UCNP in biological systems. Nanoparticle concentrations commonly used in in-vitro studies range from 1 to 100 ppm (µg/mL)^19,20,56^. Verma et al. showed that fluoride concentrations of 120 ppm decreased cell viability up to 60%^57^. Although fluoride is generally considered toxic at concentrations above 15 ppm^41,42^, long term exposure (up to 5 days) to non-lethal fluoride concentrations as low as 5 ppb of F^-^ were shown to decrease cellular DNA synthesis and a F^-^ concentration of 38 ppb almost completely inhibited it^41^.

Considering that the concentration of released fluoride ions in our experiments was in the range of 1–5 ppm after incubation for 72 h in water for all samples studied here (see SI, Tables S5-S6), this confirms the concerns raised regarding the biocompatibility of bare, citrate- and PAA-stabilized UCNP.

### Shelling with meso- and microporous silica

**Figure 3:**
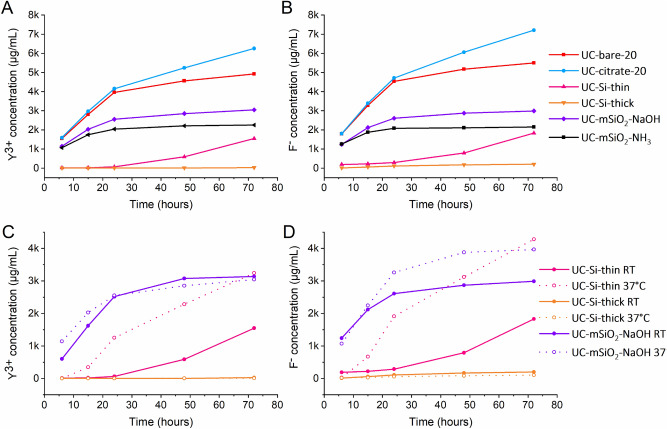
Comparison of the dissolution behavior of 30 nm bare UCNP (red line) to 30 nm UCNP with different silica coatings (see legend) in water at room temperature for up to 72 h and a UCNP concentration of 50 mg/L. (**A)** shows the Y^3+^ concentrations obtained by ICP/OES and (**B)** shows the concentrations of F^−^ obtained by ISE. (**C)** shows released Yttrium ion concentrations obtained by ICP/OES and (**D)** shows released fluoride ion concentrations measured by ISE. The lines are only a guide to the eye.

Two different silica shells were applied, a mesoporous and a microporous silica shell. A mesoporous coating yields a highly porous silica surface which is beneficial for many life science applications^10,15,45,58,59^, while a microporous silica shell is expected to provide a better surface protection, and consequently, a lower tendency towards core nanoparticle dissolution. For the preparation of the mesoporous silica shell, ammonia solution or sodium hydroxide were used as basic catalysts for the hydrolysis of TEOS^60^. For this study, a thin (10 nm) and a thick (73 nm) microporous silica shells as well as a mesoporous silica shell (23 nm) were applied to 30 nm UCNP and the particles were then aged under similar conditions as previously employed for UCNP surface-stabilized with organic ligands (see Fig. 3A,B). UC-bare-30 were used as a control (see Fig. 3, red curve). As shown in Fig. 3, shelling with a mesoporous silica shell decreased UCNP dissolution by 55% for UC-mSiO_2_–NH_3_ and 39% for UC-mSiO_2_–NaOH relative to the bare control sample. The number of ions released from UCNP coated with a thick microporous silica shell was negligible, which clearly demonstrates that a thicker silica shell provided a better protection for the UCNP against dissolution.

### Effect of temperature

Subsequently, UCNP dissolution was studied at room temperature and at 37 °C mimicking in-vitro and in-vivo conditions for the more stable silica-shelled UCNP. Aging of the silica coated samples at body temperature was used to assess the efficiency of silica coating in dissolution prevention or inhibition for cellular and bioimaging studies. As summarized in Fig. 3C,D, dissolution is typically enhanced at a higher temperature. As expected, the dissolution of the UCNP coated with a thin microporous or a mesoporous shell increased with increasing temperature while UCNP coated with a thick microporous silica shell remained stable also at 37 °C.

### Effect of the chemical composition of the aqueous medium

**Figure 4:**
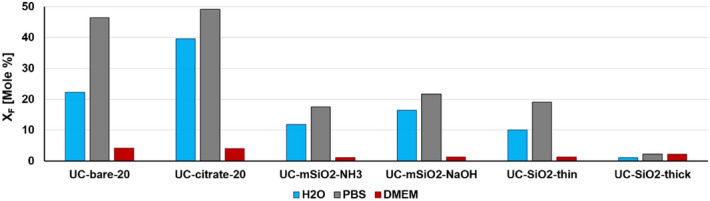
Mole fractions of fluoride ions released from UCNP (50 mg/L) with different surface chemistries upon aging for 72 h in different solvents: water at RT (H_2_O, blue bar), PBS at RT (PBS, grey bar), and cell culture medium at 37 °C (DMEM, dark red bar).

We assessed the stability of our ligand-stabilized and silica coated UCNP in water, PBS and in DMEM (which is used in cell culture experiments). The results, summarized in Fig. 4, reveal the strongest dissolution of UCNP in PBS, as indicated by the increased amount of released fluoride ions. This is ascribed to the high tendency of lanthanide ions to form stable complexes with phosphates and agrees well with the findings of Lisjak et. al^61^. However, we could not detect lanthanide ions in the supernatants after UCNP aging in PBS. This finding is ascribed to the low solubility and precipitation of the lanthanide phosphate complexes formed. The most intriguing effects were observed for aging UCNP with different surface chemistries in DMEM, which contains a mixture of biomolecules such as amino acids, sugars, vitamins, and salts (Table S2, SI). In this case, only very small amounts of fluoride ions were released for all UCNP studied as shown in Fig. 4. This strongly enhanced UCNP stability is ascribed to the formation of a protective corona of adsorbed biomolecules on the UCNP surface, similar to the formation of a protein corona observed for many nanoparticle systems in plasma and in intracellular fluids which is known to significantly affect nanoparticle toxicity^38–43^.

### Fluorescence monitoring of UCNP aging

**Figure 5:**
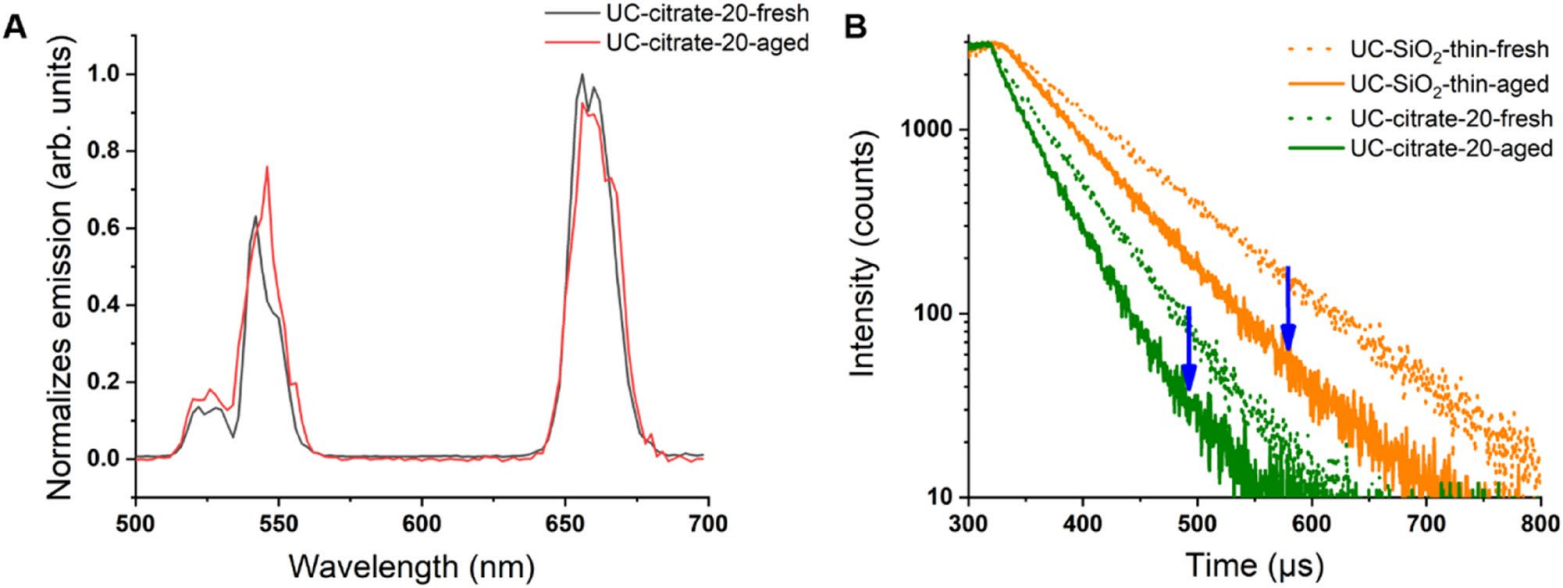
(**A)** Normalized UCL emission spectra of fresh (black line) and aged (red line) 20 nm citrate coated UCNP excited at 976 nm and normalized to the red emission at 655 nm. (**B)** Decay kinetics of the Yb^3+^ luminescence of fresh (orange, dotted line) and aged (orange, solid line) UCNP coated with a thin silica shell and stabilized with citrate (green, dotted and solid lines) excited at 976 nm and detected at 1010 nm. For the time-resolved studies, an excitation power density of 75 W/cm^2^ was used. Aged samples were incubated in water for 48 h, collected by centrifugation, and then redispersed in water; fresh samples were dispersed in water and measured immediately.

**Figure 6:**
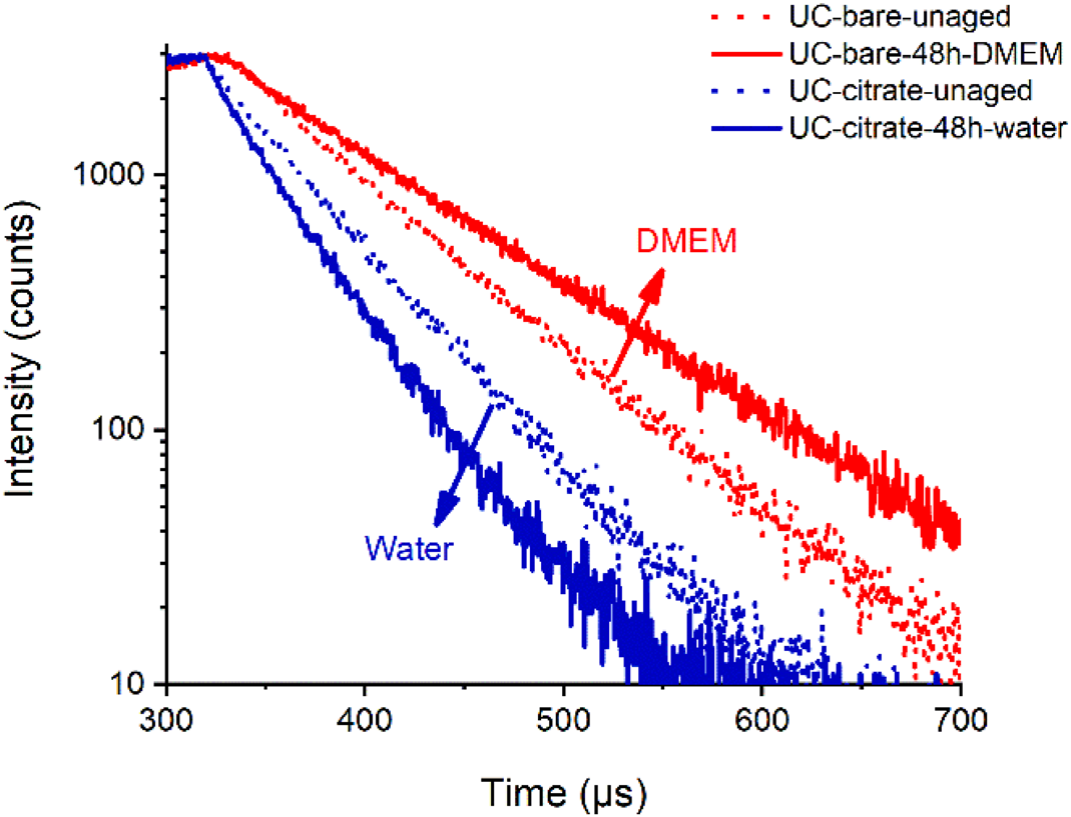
Decay curves of fresh (red, dotted line) and DMEM-aged (red, solid line, red) 20 nm bare UCNP and fresh (dotted line, blue) and H_2_O-aged (straight line, blue) 20 nm UC-citrate nanoparticles. The aged UCNP were collected and redispersed in milli-Q water for the measurement of the decay kinetics. The Yb^3+^ emission was excited at 976 nm and detected at 1010 nm.

Since UCNP dissolution causes a decrease in UCNP size^55^, and the intensity and relative spectral distribution of UCL and particularly the luminescence decay kinetics of the Yb^3+^ emission depend on UCNP size and microenvironment^23,25,34,62,63^, luminescence measurements were used to monitor UCNP dissolution. Thereby, we examined the UCL features and the downshifted Yb^3+^ emission of the fresh and aged UCNP samples varying in core size, surface chemistry, and UCNP concentration using identical instrument settings (particularly matching excitation power densities). The results of the lifetime measurements are summarized in Fig. 5, and the lifetimes are given in the SI (Table S8). As shown in Fig. 5A, aging of UCNP in water has only a small influence on the relative spectral distribution of the normalized emission spectra obtained at the same excitation power density and also on the red-to-green ratio of the Er^3+^ emission bands. Therefore, for monitoring the dissolution effects, we focused solely on measurements of the luminescence lifetimes of the Yb^3+^ and the green Er^3+^ emission excited via energy transfer upconversion (ETU) as these parameters have been previously identified to respond sensitively to aging-induced changes^62,64^. Moreover, the decay kinetics are independent of UCNP concentration and are more easily accessible for very dilute samples. Figure 5B highlights the noticeable dilution-induced decrease in the luminescence lifetimes of the Yb^3+^ of the aged UCNP compared to the fresh UCNP in water, exemplarily for 20 nm-sized UCNP coated with citrate and a thin microporous silica shell, respectively. The variation in the lifetimes of the aged UCNP coated with a thin silica shell compared to the lifetimes of their unaged counterparts suggests particles dissolution. Apparently, a 10 nm silica shell is not sufficient for inhibiting particle dissolution. Similar changes in luminescence decay kinetics of Yb^3+^ reflecting the trends observed in the previously discussed analytical studies were obtained for the other UCNP samples except for aging in DMEM. Here, time-resolved luminescence measurements with UCNP, as shown exemplarily for bare and citrate stabilized UCNP in Fig. 6, revealed a considerable increase in the Yb^3+^ luminescence lifetime. This suggests a shielding of the UCNP surface for the DMEM-aged samples, thereby supporting the hypothesis of the formation of a protective bio-corona on the surface of the UCNP.

### XPS and MS study of the organic surface layer formed in DMEM

**Figure 7:**
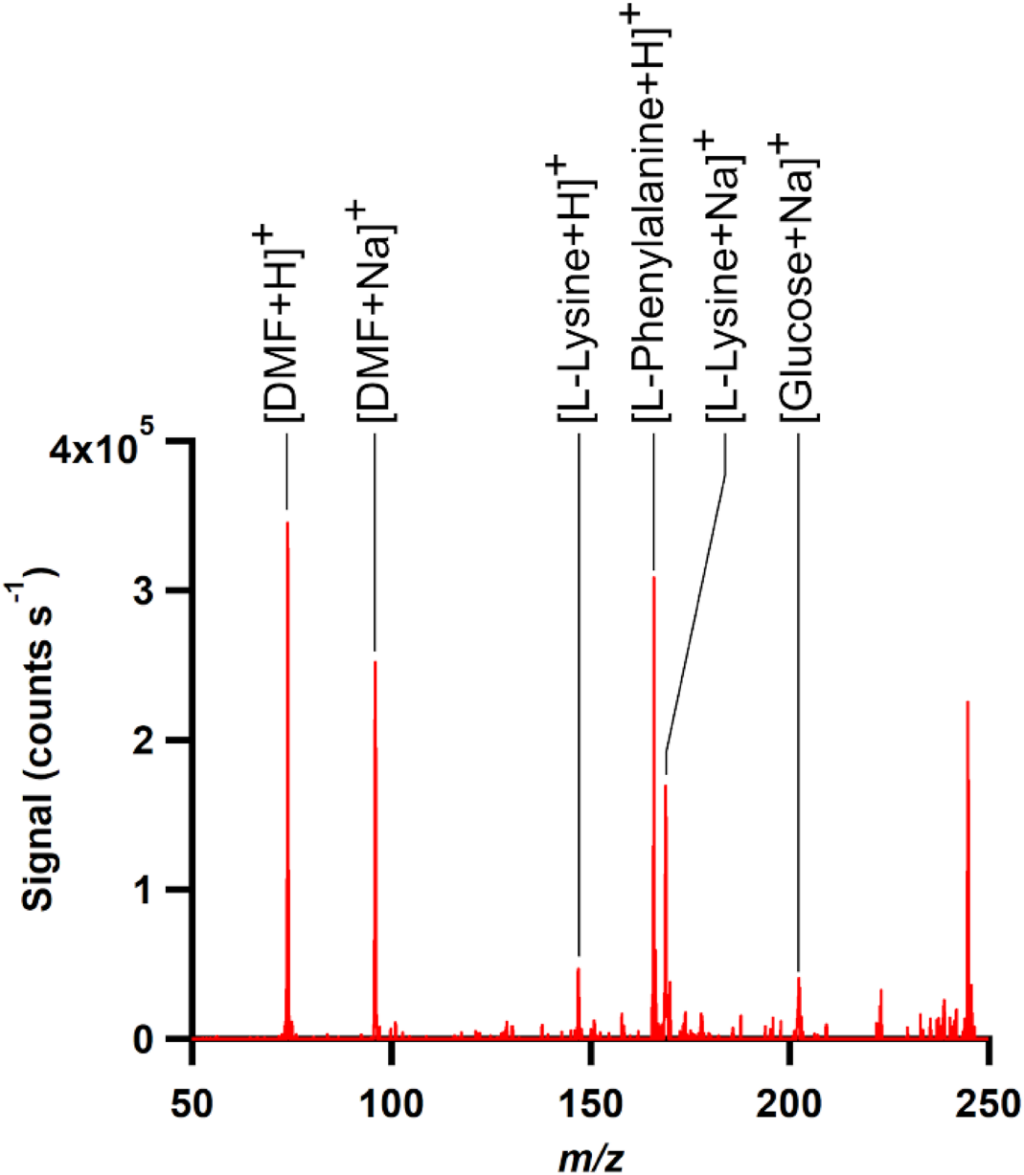
Mass spectrum of the aqueous solution containing components of the “corona” layer that results from incubating UCNP in DMEM.

To gain more information on the DMEM-induced surface modification and the DMEM components on the UCNP surface, we subsequently analysed DMEM-aged samples with XPS and MS. First, XPS measurements of UC-bare-20 nanoparticles before and after incubation in DMEM were performed to study changes in the surface chemistry. The particles were incubated in DMEM, isolated by centrifugation, redispersed in 10 µL of water, cast on a silicon wafer, and left to dry (see also Materials and Methods section). While the transfer to water seems to cause partial dissociation of the organic layer from the UCNP surface as revealed by lifetime measurements shown in the SI (Figure S9), parts of the adsorbed layer of DMEM constituents still remain on the particle surface as revealed by the still slightly prolongated decay behavior of the DSL compared to the starting UCNP (see SI, Figure S9, comparison of green and blue decay curves). Hence, the organic components detected by XPS after UCNP drying are associated with the DMEM-aged UCNP sample. The XPS measurements after incubation in DMEM showed—besides the presence of inorganic components from the UCNP themselves (Y^3+^, F^−^ and Na^+^ ions)—also the presence of new nitrogen-containing species and changes in the relative amounts of carbon and oxygen species. This is ascribed to the adsorption of organic molecules on the surface of the UCNP (see Tables S10 and S11, SI). To further investigate the DMEM components adsorbed on the UCNP surface, we also performed MS analyses. Again, we incubated a sample of UC-bare-20 nanoparticles in DMEM in an identical procedure to that used for preparing the XPS sample and isolated the DMEM-surface modified particles by centrifugation. Then, the molecules adsorbed on the UCNP surface were desorbed by placing the UCNP in phosphate buffer and the mass spectra of the resulting solution (containing the molecules desorbed in phosphate buffer) were measured. Based on the well-known chemical context of DMEM, we could assign the peaks found in the measured sample by interpreting their *m/z* with respect to the possible DMEM components shown in the SI (Table S2). One should bear in mind again that all organic compounds detected in the solution by MS had to come either from DMEM components that were introduced together with the sample and hence previously adsorbed on the UCNP surface, or from traces of oleic acid or DMF that remained associated with the nanoparticle surface after the ligand exchange process. MS analysis of the supernatant led to six main peaks as shown in Fig. 7. The peaks at *m/z* 148 and *m/z* 170 were assigned to [Lysine + H]^+^ and [Lysine + Na]^+^, the peak at *m/z* 166 to [Phenylalanine + H]^+^, and the peak at *m/z* 220 to [Glucose + Na]^+^ (isobar with [*myo*-Inositol + Na]^+^), respectively. Likely candidates for the peaks at *m/z* 74 and 96 are [Dimethylformamide + H]^+^ and [Dimethylformamide + Na]^+^, respectively.

## Conclusion and outlook

We present a systematic study on the aging and dissolution of 20 nm- and 30 nm-sized β-NaYF4:Yb,Er upconversion nanoparticles (UCNP), surface-stabilized with the coordinatively bound ligands citrate and polyacrylic acid (PAA) or with mesoporous or microporous silica shells of different thickness in water of neutral pH, phosphate buffer saline (PBS) solution, and the cell culture medium DMEM at room temperature and 37 °C.

Particle dissolution was quantified with a fluoride-selective electrode and inductively coupled plasma optical emission spectroscopy. Our results revealed that a reduced particle size, an increased temperature, and a decreased UCNP concentration, particularly for UCNP with coordinatively bound ligands, all favour UCNP dissolution. These findings agree well with the results from previous studies. Dissolution was more pronounced in PBS containing lanthanide-binding phosphate anions compared to water. Silica shelling of the UCNP surface considerably improved particle stability in water and in PBS under all conditions assessed, and a sufficiently thick microporous silica shell proved to be able to protect UCNP from dissolution. Here, further studies are required to optimize the thickness of the silica shell regarding optimum protection with minimum thickness, as the size of a nanoparticle can be an important parameter for many biological and bioanalytical applications.

Surprisingly, DMEM led to a strong stabilization of all UCNP. This was ascribed to the formation of a surface-shielding bio-corona of adsorbed DMEM constituents that prevented the release of detectable amounts of fluoride and lanthanide ions. The formation of such a bio-corona was supported by an increase in the lifetime of the downshifted Yb^3+^ emission which is known to respond strongly to the presence of nearby surface quenchers containing high energy vibrations, such as water molecules. XPS measurements revealed the presence of a high percentage of carbon, oxygen, and nitrogen on the UCNP surface, originating from organic DMEM compounds. Mass spectrometry measurements further suggested that phenylalanine, lysine, and glucose are the main DMEM constituents that adsorb on the surface of the UCNP. Further in depth-studies of the formation and identity of this corona layer, as well as its utilization to engineer new surface coatings based on similar motifs will be done in the future for developing protective biocompatible coatings.

Moreover, our findings concerning the formation of a surface protective layer originating from DMEM on the UCNP have a large impact on the interpretation of cytotoxicity studies of nanoparticles that normally use nanoparticles dispersed in cell culture medium prior to their incubation with cells. For example, this could possibly explain the low cytotoxicity of UCNP previously reported in the literature, despite the fact that the same particles show non-negligible dissolution and release of potentially harmful ions such as Fluoride and heavy metals in water and buffer up to levels that might cause cytotoxic effects. This suggests that further in-vitro and in-vivo studies are required to draw conclusions about the UCNP cytotoxicity.

## Supplementary information

Supplementary Information

## References

[1] Haase M, Schäfer H (2011). Upconverting nanoparticles. Angew. Chem. Int. Ed. Engl..

[2] Wang F, Banerjee D, Liu Y, Chen X, Liu X (2010). Upconversion nanoparticles in biological labeling, imaging, and therapy. Analyst.

[3] Chen G, Qiu H, Prasad PN, Chen X (2014). Upconversion nanoparticles: design, nanochemistry, and applications in theranostics. Chem. Rev..

[4] Wang F, Liu X (2009). Recent advances in the chemistry of lanthanide-doped upconversion nanocrystals. Chem. Soc. Rev..

[5] Auzel F (2004). Upconversion and Anti-Stokes Processes with f and d Ions in Solids. Chem. Rev..

[6] Qian H-S, Zhang Y (2008). Synthesis of hexagonal-phase core-shell NaYF4 nanocrystals with tunable upconversion fluorescence. Langmuir.

[7] Resch-Genger U, Gorris HH (2017). Perspectives and challenges of photon-upconversion nanoparticles - Part I: routes to brighter particles and quantitative spectroscopic studies. Anal. Bioanal. Chem..

[8] Heng Qin DW, Juna S, Xiangyu X, Mary R, Fang X (2018). Tuning the upconversion photoluminescence lifetimes of NaYF4:Yb3+, Er3+ through lanthanide Gd3+ doping. Sci. Rep..

[9] Gorris HH, Resch-Genger U (2017). Perspectives and challenges of photon-upconversion nanoparticles - Part II: bioanalytical applications. Anal. Bioanal. Chem..

[10] Liu J, Li C, Li F (2011). Fluorescence turn-on chemodosimeter-functionalized mesoporous silica nanoparticles and their application in cell imaging. J. Mater. Chem..

[11] Alonso-de Castro S (2019). Functionalizing NaGdF4:Yb, Er upconverting nanoparticles with bone-targeting phosphonate ligands: imaging and in vivo biodistribution. Inorganics.

[12] Del Rosal B, Jaque D (2019). Upconversion nanoparticles for in vivo applications: limitations and future perspectives. Methods Appl Fluoresc..

[13] Generalova AN (2016). PEG-modified upconversion nanoparticles for in vivo optical imaging of tumors. RSC Adv..

[14] Park YI, Lee KT, Suh YD, Hyeon T (2015). Upconverting nanoparticles: a versatile platform for wide-field two-photon microscopy and multi-modal in vivo imaging. Chem. Soc. Rev..

[15] Liu J, Bu W, Pan L, Shi J (2013). NIR-triggered anticancer drug delivery by upconverting nanoparticles with integrated azobenzene-modified mesoporous silica. Angew. Chem. Int. Ed. Engl..

[16] Peng J (2013). Polyphosphoric acid capping radioactive/upconverting NaLuF4:Yb, Tm,153Sm nanoparticles for blood pool imaging in vivo. Biomaterials.

[17] Oliveira H (2019). Critical considerations on the clinical translation of upconversion Nanoparticles (UCNPs): recommendations from the European Upconversion Network (COST Action CM1403). Adv. Healthc. Mater..

[18] Abdul Jalil R, Zhang Y (2008). Biocompatibility of silica coated NaYF(4) upconversion fluorescent nanocrystals. Biomaterials.

[19] Gnach A, Lipinski T, Bednarkiewicz A, Rybka J, Capobianco JA (2015). Upconverting nanoparticles: assessing the toxicity. Chem. Soc. Rev..

[20] Wysokinska E (2019). Toxicity mechanism of low doses of NaGdF(4):Yb(3+), Er(3+) upconverting nanoparticles in activated macrophage cell lines. Biomolecules.

[21] Feng W, Zhu X, Li F (2013). Recent advances in the optimization and functionalization of upconversion nanomaterials for in vivo bioapplications. NPG Asia Mater..

[22] Li Z, Zhang Y, Jiang S (2008). Multicolor core/shell-structured upconversion fluorescent nanoparticles. Adv. Mater..

[23] Wang F (2010). Simultaneous phase and size control of upconversion nanocrystals through lanthanide doping. Nature.

[24] Andresen E, Resch-Genger U, Schaferling M (2019). Surface modifications for photon-upconversion-based energy-transfer nanoprobes. Langmuir.

[25] Wilhelm S (2015). Water dispersible upconverting nanoparticles: effects of surface modification on their luminescence and colloidal stability. Nanoscale.

[26] Himmelstoss SF, Hirsch T (2019). A critical comparison of lanthanide based upconversion nanoparticles to fluorescent proteins, semiconductor quantum dots, and carbon dots for use in optical sensing and imaging. Methods Appl. Fluoresc..

[27] Sedlmeier A, Gorris HH (2015). Surface modification and characterization of photon-upconverting nanoparticles for bioanalytical applications. Chem. Soc. Rev..

[28] Himmelstoß SF, Hirsch T (2019). Long-term colloidal and chemical stability in aqueous media of NaYF4-type upconversion nanoparticles modified by ligand-exchange. Particle Particle Syst. Charact..

[29] Estebanez N, Gonzalez-Bejar M, Perez-Prieto J (2019). Polysulfonate cappings on upconversion nanoparticles prevent their disintegration in water and provide superior stability in a highly acidic medium. ACS Omega.

[30] Plohl O (2017). Amphiphilic coatings for the protection of upconverting nanoparticles against dissolution in aqueous media. Dalton Trans..

[31] Dutta J, Rai VK (2019). APTES modified GO-PEI-Er3+/Yb3+: NaYF4 upconverting nanoparticles hybrid film-based optical pH sensor and NIR photoelectric response. IEEE Sens. J..

[32] 32Ruibin Li, Z. J., Juyao, D., Chong Hyun, C., Xiang, W., Bingbing, S., Meiying, W., Yu-Pei, L., Jeffrey, I. Z., Andre, E. N., Tian, X. Enhancing the imaging and biosafety of upconversion nanoparticles through phosphonate coating. *ACS Nano* **9** (2015). 10.1021/acsnano.5b00439 PMC4415359 25727446

[33] Mondal M, Rai VK, Srivastava C (2017). Influence of silica surface coating on optical properties of Er 3+ -Yb 3+ :YMoO 4 upconverting nanoparticles. Chem. Eng. J..

[34] Arppe R (2015). Quenching of the upconversion luminescence of NaYF(4):Yb(3)(+), Er(3)(+) and NaYF(4):Yb(3)(+), Tm(3)(+) nanophosphors by water: the role of the sensitizer Yb(3)(+) in non-radiative relaxation. Nanoscale.

[35] Wurth C (2017). Excitation power dependent population pathways and absolute quantum yields of upconversion nanoparticles in different solvents. Nanoscale.

[36] Wurth C, Fischer S, Grauel B, Alivisatos AP, Resch-Genger U (2018). Quantum yields, surface quenching, and passivation efficiency for ultrasmall core/shell upconverting nanoparticles. J. Am. Chem. Soc..

[37] Xiao Q (2014). Rational design of a thermalresponsive-polymer-switchable FRET system for enhancing the temperature sensitivity of upconversion nanophosphors. Nanoscale.

[38] Itoh Hisako HH (1984). Tsuchiya masashi, suzuki yasuo, asano yasukazu determination of solubility products of rare earth fluorides by fluoride ion-selective electrode. Bull. Chem. Soc. Jpn..

[39] Barbier O, Arreola-Mendoza L, Del Razo LM (2010). Molecular mechanisms of fluoride toxicity. Chem. Biol. Interact..

[40] 40Palmer, R, J. & Stevens, J. B. Cytotoxicity of the rare earth metals cerium, lanthanum, and neodymium in vitro: comparisons with cadmium in a pulmonary macrophage primary culture system. *Environ. Res.* **43** (1987). 10.1016/s0013-9351(87)80066-x 3582304

[41] Chang YC, Chou MY (2001). Cytotoxicity of fluoride on human pulp cell cultures in vitro. Oral Surg. Oral Med. Oral Pathol. Oral Radiol. Endod..

[42] Salomao PMA (2017). The cytotoxic effect of TiF4 and NaF on fibroblasts is influenced by the experimental model, fluoride concentration and exposure time. PLoS ONE.

[43] Lisjak D, Plohl O, Ponikvar-Svet M, Majaron B (2015). Dissolution of upconverting fluoride nanoparticles in aqueous suspensions. RSC Adv..

[44] Radunz S (2018). Evolution of size and optical properties of upconverting nanoparticles during high-temperature synthesis. J. Phys. Chem. C.

[45] Li C, Liu J, Alonso S, Li F, Zhang Y (2012). Upconversion nanoparticles for sensitive and in-depth detection of Cu2+ ions. Nanoscale.

[46] Dong A (2011). A generalized ligand-exchange strategy enabling sequential surface functionalization of colloidal nanocrystals. J. Am. Chem. Soc..

[47] Carron S (2015). Assembly of near infra-red emitting upconverting nanoparticles and multiple Gd(III)-chelates as a potential bimodal contrast agent for MRI and optical imaging. Dalton Trans..

[48] Juan J, Cheng L, Shi M, Liu Z, Mao X (2015). Poly-(allylamine hydrochloride)-coated but not poly(acrylic acid)-coated upconversion nanoparticles induce autophagy and apoptosis in human blood cancer cells. J. Mater. Chem. B.

[49] Kembuan C, Saleh M, Rühle B, Resch-Genger U, Graf C (2019). Coating of upconversion nanoparticles with silica nanoshells of 5–250 nm thickness. Beilstein J. Nanotechnol..

[50] 50In 15472:2010 (Geneva, 2010).

[51] Hesse R, Denecke R (2011). Improved Tougaard background calculation by introduction of fittable parameters for the inelastic electron scattering cross-section in the peak fit of photoelectron spectra with UNIFIT 2011. Surf. Interface Anal..

[52] Song L, You Y, Evans-Nguyen T (2019). Surface acoustic wave nebulization with atmospheric-pressure chemical ionization for enhanced ion signal. Anal. Chem..

[53] You Y, Badal SP, Shelley JT (2019). Automatic analyte-ion recognition and background removal for ambient mass-spectrometric data based on cross-correlation. J. Am. Soc. Mass Spectrom..

[54] Dukhno O (2018). Time-dependent luminescence loss for individual upconversion nanoparticles upon dilution in aqueous solution. Nanoscale.

[55] Lahtinen S (2016). Disintegration of hexagonal NaYF4:Yb3+, Er3+ upconverting nanoparticles in aqueous media: the role of fluoride in solubility equilibrium. J. Phys. Chem. C.

[56] Guller AE (2015). Cytotoxicity and non-specific cellular uptake of bare and surface-modified upconversion nanoparticles in human skin cells. Nano Res..

[57] Verma A, Ali D, Pathak AK (2017). Fluoride induces DNA damage and cytotoxicity in human hepatocellular carcinoma cells. Toxicol. Environ. Chem..

[58] Argyo C, Weiss V, Bräuchle C, Bein T (2013). Multifunctional mesoporous silica nanoparticles as a universal platform for drug delivery. Chem. Mater..

[59] Qian HS, Guo HC, Ho PC, Mahendran R, Zhang Y (2009). Mesoporous-silica-coated up-conversion fluorescent nanoparticles for photodynamic therapy. Small.

[60] Li Z, Jia L, Li Y, He T, Li X-M (2015). Ammonia-free preparation of Ag@SiO 2 core/shell nanoparticles. Appl. Surf. Sci..

[61] Lisjak D, Plohl O, Vidmar J, Majaron B, Ponikvar-Svet M (2016). Dissolution mechanism of upconverting AYF4:Yb, Tm (A = Na or K) nanoparticles in aqueous media. Langmuir.

[62] Plohl O (2017). Optically detected degradation of NaYF4:Yb, Tm-based upconversion nanoparticles in phosphate buffered saline solution. Langmuir.

[63] Wang F, Wang J, Liu X (2010). Direct evidence of a surface quenching effect on size-dependent luminescence of upconversion nanoparticles. Angew. Chem. Int. Ed. Engl..

[64] Andresen E, Wurth C, Prinz C, Michaelis M, Resch-Genger U (2020). Time-resolved luminescence spectroscopy for monitoring the stability and dissolution behaviour of upconverting nanocrystals with different surface coatings. Nanoscale.

