# Supplementary material for: Assessing the protective effects of different surface coatings on NaYF4:Yb3+, Er3+ upconverting nanoparticles in buffer and DMEM
Source: Sci Rep. 2020 Nov 9;10:19318. doi: 10.1038/s41598-020-76116-z (PMC7652843; doi:10.1038/s41598-020-76116-z)
Supplement: Supplementary file 1 — Supplementary Information [file 41598_2020_76116_MOESM1_ESM.pdf]

Supplementary Information of

Assessing the Protective Effects of Different Surface Coatings on $NaYF_4:Yb^{3+}$, Er^{3+} Upconverting Nanoparticles in Buffer and DMEM

Maysoon I. Saleh[a,b], Bastian Rühle[a], Shu Wang[a,b], Jörg Radnik[c], Yi You[d] and Ute Resch-Genger[a,*]

[a.] Federal Institute for Materials Research and Testing, Division 1.2 Biophotonics, Richard-Willstätter-Str. 11, 12489 Berlin, Germany.
Email: ute.resch@bam.de

[b.] Institut für Chemie und Biochemie, Freie Universität Berlin, Takustrasse 3, D-14195 Berlin, Germany.

[c.] Federal Institute for Materials Research and Testing, Division 6.1, Unter den Eichen 44-46, 12203 Berlin, Germany.

[d.] Federal Institute for Materials Research and Testing, Division 6.3, structural analysis, Richard-Willstätter-Str. 11, 12489 Berlin, Germany.

Table of Contents

1. Synthesis of Upconversion Nanoparticles (UCNP) 2
2. Surface Modification of UCNP 3
3. Components of DMEM 5
4. Characterization of UCNP (TSEM, XRD, DLS, Absorption and Emission) 6
5. Analytical Measurements for the Detection of the Released Ions 9
6. Luminescence Lifetime Measurements 12
7. X-ray Photoelectron Spectroscopic Measurements Results 15
8. References 16

1. Synthesis of Upconversion Nanoparticles (UCNP)

Upconversion nanoparticles (UCNP) were prepared via a thermal decomposition method, with some modification to published procedures.[1-3] This synthesis was done in two steps: Preparation of the nanoparticle precursors, and then high temperature growth of the nanoparticles.

1. Synthesis of the precursor: In a round-bottom flask, sodium oleate (9.146 g, 30.00 mmol) was dissolved in a mixture of 20 ml of H_2O and 30 mL of ethanol. In a centrifuge tube YCl_36H_2O (2.442 g, 8.04 mmol), $YbCl_3.6H_2O$ (0.716 g, 1.84 mmol) and $ErCl_3 \cdot 6H_2O$ (0.078 g, 0.2 mmol) were dissolved in a mixture of 30 mL of H_2O, 70 mL of ethanol and 70 mL of cyclohexane. The two solutions were mixed and heated under refluxed for 4h. After cooling the resulting yellow homogeneous solution to RT, the product was dried and collected using a rotary evaporator.

2. Synthesis of $NaYF_4$:20%Yb^{3+},2%Er^{3+} (UCNP): The lanthanide precursor was dissolved in a solution of 150 mL of 1-octadecene and 70 mL of oleic acid. The solution was heated to 100°C to degas, and then allowed to cool to 50°C under argon. Then, 30 mL of a methanolic solution containing NaOH (1.00 g, 25.000 mmol) and NH_4F (1.48 g, 39.960 mmol) were added slowly to the precursor and the reaction mixture was stirred for 30 min at 50°C to ensure that all the fluorides were dissolved. The solution was heated to 100°C to remove methanol, then it was heated to 300°C and the reaction was kept at this temperature for 2.0 h under an inert atmosphere of Ar. After that, the reaction solution was allowed to cool, and the nanoparticles were precipitated with ethanol and isolated by centrifugation.

Modification to the first procedure

Throughout the course of work, the synthesis of UCNP was modified.[4,5] The modified approach also produced monodisperse, bright nanoparticles, but the reproducibility in obtaining the desired size of the nanoparticles was improved by controlling the reaction time based on the use of a portable 980 nm diode laser. Both procedures shared the first step of preparing the precursor, with some changes to the formation of the nanoparticles in the second procedure.

The dry precursor was dissolved in 70 mL of oleic acid and 150 mL of 1-octadecene and heated to 160 °C under argon. After 30 minutes, a clear solution was formed and then the reaction solution was degassed under vacuum using a Schlenk line. The reaction mixture was cooled to room temperature and then 35 mL of a methanolic solution of NaOH (13 mmol) and NH_4F (20 mmol) were added slowly. The suspension was stirred for 30 minutes at 120 °C under a flow of argon and then heated to 325°C (a reflux condenser was connected at the very high temperature to avoid any possible evaporation of the solvents).

The reaction was then monitored by a 980 nm laser. A green emission was observed within 10 minutes after reaching 325°C. In the beginning of the reaction, weakly emitting α-particles will form which then grow further through Ostwald ripening and form β-particles that show a very intense green emission that can readily be observed with the naked eye after 15 minutes at 325°C. At this point, the mixture was cooled to room temperature, the oleate-capped UCNP were precipitated by addition of an appropriate amount of ethanol, and the dispersion was centrifuged at 1000 g for 5 minutes. The product was redispersed three times in chloroform and reprecipitated in ethanol, and then washed with cyclohexane and acetone. The purified UCNP were stored in cyclohexane.

2. Surface Modification of UCNP

Coating of UCNP with a Mesoporous Silica Shell

a. Preparation of CTAB-stabilized UCNP

The procedure for coating the UCNP with a mesoporous silica shell was based on an approach reported in literature.[6] In this coating process, the UCNP are first transferred to water by stabilization with CTAB (cetyltrimethylammonium bromide) prior to silica growth. 0.4 g of CTAB was dissolved in 80 mL of water, then 2 mL of UCNP suspension in cyclohexane (20 mg/mL) was added to the aqueous CTAB solution keeping the total concentration of UCNP in water at 0.5 g/mL. The mixture was stirred for 48 h at room temperature to evaporate cyclohexane and obtain the CTAB-stabilized UCNP.

b. Coating of UCNP with a mesoporous silica shell using different basic catalysts:

For the coating process, 20 mL of water, 3 mL of EtOH and 24 µL of ammonia solution (25% w/w) were mixed in a 100 mL flask. Then, 10 mL of the CTAB-stabilized UCNPs (see above) were added to the mixture and heated to 70°C under stirring at 600 rpm. Subsequently, a mixture of 75 µL of TEOS in 4 mL of ethanol was added by the aid of a peristaltic pump (addition rate 0.5mL/min). The reaction was kept at 70°C for 1 h. The resulting UCNP@mSiO_2 nanoparticles were washed three times with ethanol. For the washing step, ethanol was added to the dispersion to sediment the nanoparticles and then the dispersion was centrifuged at 1108 g for 30 min.

Finally, the surfactant (CTAB) was removed from the silica shell by an ion exchange process. The mesoporous silica coated upconversion nanoparticles (UCNP@mSiO_2) were added to a solution of 50 mL of ethanol and 0.3 g of NH_4NO_3 and kept at 60°C for 2 h. The resulting UCNPs@mSiO_2 were then washed by centrifugation and stored in ethanol.

For the sample UC-mSiO_2-NaOH, the same procedure was used but 1.5 mL of 2 M sodium hydroxide was used instead of ammonia solution.

Coating of UCNP with a microporous silica shell

A microporous silica shell was synthesized using an oil-in-water microemulsion synthesis. In a centrifuge tube, upconversion nanoparticles in cyclohexane (4.74 mL, 6.6 mg/ml), 5.26 mL of cyclohexane and 0.6 mL of Igepal CO-520 were mixed by shaking, then 0.142 mL of ammonia solution (25% w/w) were added, and the mixture was mixed again by shaking the tube. The silica precursor, tetraethyl orthosilicate (TEOS, 0.142 mL; 0.637 mmol) was added into the mixture, and the mixture was kept on a vortex shaker overnight. Upconversion nanoparticles with a microporous silica shell were then sedimented and washed three times with ethanol by redispersion and centrifugation at 1108 g for 30 minutes.

Silica re-growth to form a thicker microporous silica shell

The re-growth of the silica shell on the surface of the upconversion nanoparticles was done in several steps following a published protocol.[7-9] First, thin silica shells were grown by oil-in-water microemulsion processes as described in the previous section. In each step, the reaction mixture was kept stirring after adding TEOS for at least 12 hours, and then the next shell was formed by adding the required amount of cyclohexane, ammonia solution, Igepal CO-520 and TEOS without purification or breaking the microemulsion. Five thin shells were applied following this procedure until the final diameter of the core/shell nanoparticles was 123 nm. At this point, the nanoparticles were sedimented by ethanol and washed three times with EtOH by centrifugation, dispersed in absolute ethanol, and further coated with silica through a modified Stöber process using published protocols with some changes. Reactants amounts are listed in Table S1:

Table S1: The amounts of reactants used to produce the microporous silica shells on the surface of the upconversion nanoparticles. All syntheses of the silica shells were performed at room temperature.

**Oil-in-water microemulsion synthesis of the silica shell**<br>Upconversion nanoparticles core diameter: 30 nm, mass: 32 mg, no. of particles: $5.45 \times 10^{+14}$					
Silica shell	Cyclohexane (mL)	Igepal (mL)	NH_3 (25%) (µL)	TEOS (µL)	Shell thickness (nm)
1	10	0.60	124	124	10
2	10	1.00	248	248	18
3	19	1.65	428	428	26
4	28	2.65	676	676	32
5	54	2.52	1420	1420	47
**Modified Stöber synthesis the silica shell**<br>UC@SiO_2 core diameter: 123 nm, mass: 96 mg, no. of particles: $4.78 \times 10^{+13}$					
Silica shell	Ethanol (mL)	NH_3 (25%) (µL)	TEOS (µL)	TEOS addition rate (µL/min)	Shell thickness (nm)
6	20	940	1169	700	73

Removal of the oleate ligand with NOBF4/DMF

Upconversion nanoparticles were treated with $NOBF_4$ to strip off the oleate ligand according to a ligand exchange strategy reported by Dong et al.[10] In detail, 10 mL of dimethylformamide (DMF) were transferred into a round bottom flask. Then, 50 mg of the oleate coated nanoparticles dispersed in 10 mL of cyclohexane were added. This resulted in a two-phase system consisting of an upper layer of cyclohexane (containing the oleate coated nanoparticles) and a lower layer of DMF. Subsequently, 120 mg (1.0 mmol) of $NOBF_4$ were added to the dispersion and sonicated for 20 minutes at 20°C until the DMF layer became turbid, indicating a phase transfer with the original hydrophobic oleic acid coating of the upconversion nanoparticles replaced by a layer of hydrophilic BF_4^-. The nanoparticles within the slightly turbid DMF phase were precipitated by chloroform and collected by centrifugation (1000 g for 10 minutes). The obtained oleate-free, BF_4^- stabilized nanoparticles were dispersed in 10 mL of DMF.

Ligand exchange with citrate

Citrate-coated upconversion nanoparticles were prepared according to a method reported by Carron et. al.[11] 0.2 M citric acid buffers with pH values of 3 and 6 were prepared. Then, 20 mg of the oleate capped nanoparticles were dispersed in 20 mL of a 0.2 M citric acid buffer (pH = 3) and stirred for three hours at 800 rpm to remove the oleic acid ligand. After that, the solution was extracted 3 times with cyclohexane and another 3 times with diethyl ether. The ligand-free nanoparticles were precipitated by adding an excess of acetone and collected by centrifugation (1000 g, 30 min). Afterwards, they were re-dispersed in 20 mL of a 0.2 M citric acid buffer (pH =6) and stirred at 800 rpm for 12h. The solution was extracted three times with cyclohexane and another three times with diethyl ether. The citrate capped nanoparticles were obtained by precipitation with acetone, followed by centrifugation (1000 g, 30 min) and then redispersed in 5 ml of absolute ethanol and stored at room temperature.

Polyacrylic acid coating on UCNP

Coating the nanoparticles with polyacrylic acid was carried out following a protocol from Juan et al.[12] 303 mg of polyacrylic acid (PAA, MW = 1800) was added to 30 mL of diethylene glycol (DEG) in a three-neck flask, and the mixture was heated to 110°C to form a clear solution. Then, 5 mL of a toluene dispersion containing 100 mg of the upconversion nanoparticles was added slowly into the PAA solution under an inert atmosphere of nitrogen while maintaining the temperature at 110 °C for 1 h.

The solution was then heated to 240°C for another 1 h. After cooling down to room temperature, the nanoparticles were precipitated by addition of ethanol, washed several times with an ethanol/water mixture (1: 1 v/v), and finally stored in absolute ethanol.

3. Components of DMEM

Table S2: Components of DMEM

**DMEM Product No. D5546**			
**Component**	**g/L**	**Component**	**g/L**
**Inorganic Salts**		L-Threonine	0.095
$CaCl_2$	0.2	L-Tryptophan	0.016
$Fe(NO_3)_3.9H_2O$	0.0001	**Amino Acids**	
$MgSO_4$	0.09767	L-Tyrosine.2Na.2H_2O	0.10379
KCl	0.4	L-Valine	0.094
$NaHCO_3$	3.7	**Vitamins**	
NaCl	6.4	Choline Chloride	0.004
NaH_2PO_4	0.109	Folic Acid	0.004
**Amino Acids**		myo -Inositol	0.0072
L-Alanyl-L-Glutamine	-	Niacinamide	0.004
L-Arginine.HCl	0.084	D-Pantothenic Acid.½Ca	0.004
L-Cystine.2HCl	0.0626	Pyridoxal.HCl	-
L-Glutamine	-	Pyridoxine.HCl	0.00404
Glycine	0.03	Riboflavin	0.0004
L-Histidine.HCl.H_2O	0.042	Thiamine.HCl	0.004
L-Isoleucine	0.105	**Other**	
L-Leucine	0.105	D-Glucose	1.0
L-Lysine.HCl	0.146	HEPES	-
L-Methionine	0.03	Phenol Red.Na	0.0159
L-Phenylalanine	0.066	Pyruvic Acid.Na	0.11
L-Serine	0.042		

4. Characterization of UCNP (TSEM, XRD, DLS, Absorption and Emission)

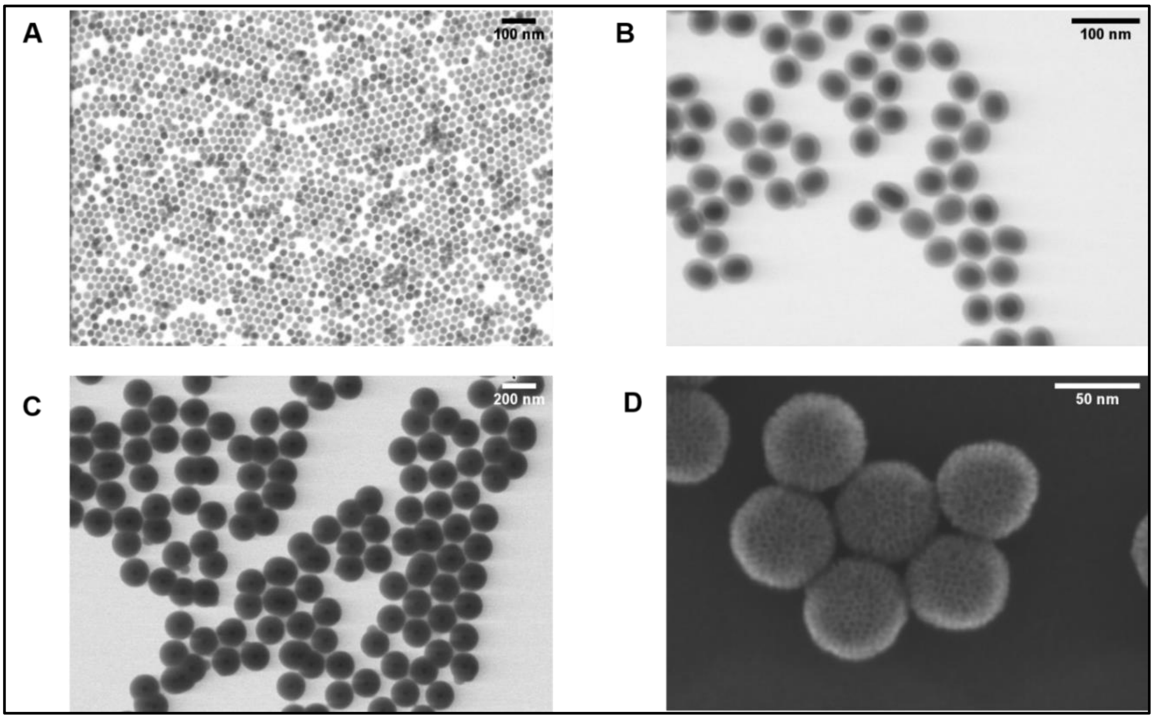

Figure S1: TSEM images of **A)** Oleate coated $NaYF_4$: 20%Yb^{3+}, 2%Er^{3+} (UCNP) redispersed in cyclohexane, average diameter: 20 nm. **B)** UCNP coated with a thin silica shell of average shell thickness of 10 nm. **C)** UCNP coated with a thick silica shell of average shell thickness of 73 nm. **D)** UCNP coated with a mesoporous silica shell of average shell thickness of 23 nm.

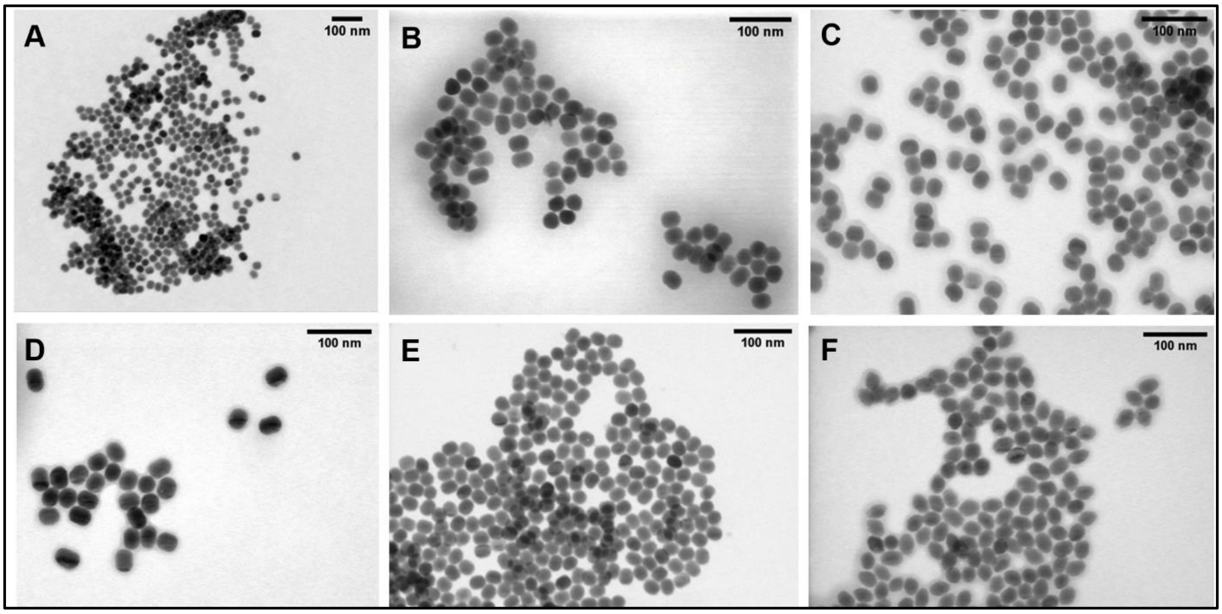

Figure S2: TSEM images of UCNP after surface modification. UCNP coated with polyacrylic acid and redispersed in water, average diameter of UCNP: 20 nm **(A)** and 30 nm **(B)**. Bare UCNP after treatment with $NOBF_4$ and redispersion in DMF, average diameter of UCNP: 20 nm **(C)** and 30 nm **(D)**. UCNP coated with citrate and redispersed in water, average diameter of UCNP: 20 nm **(E)** and 30 nm **(F)**.

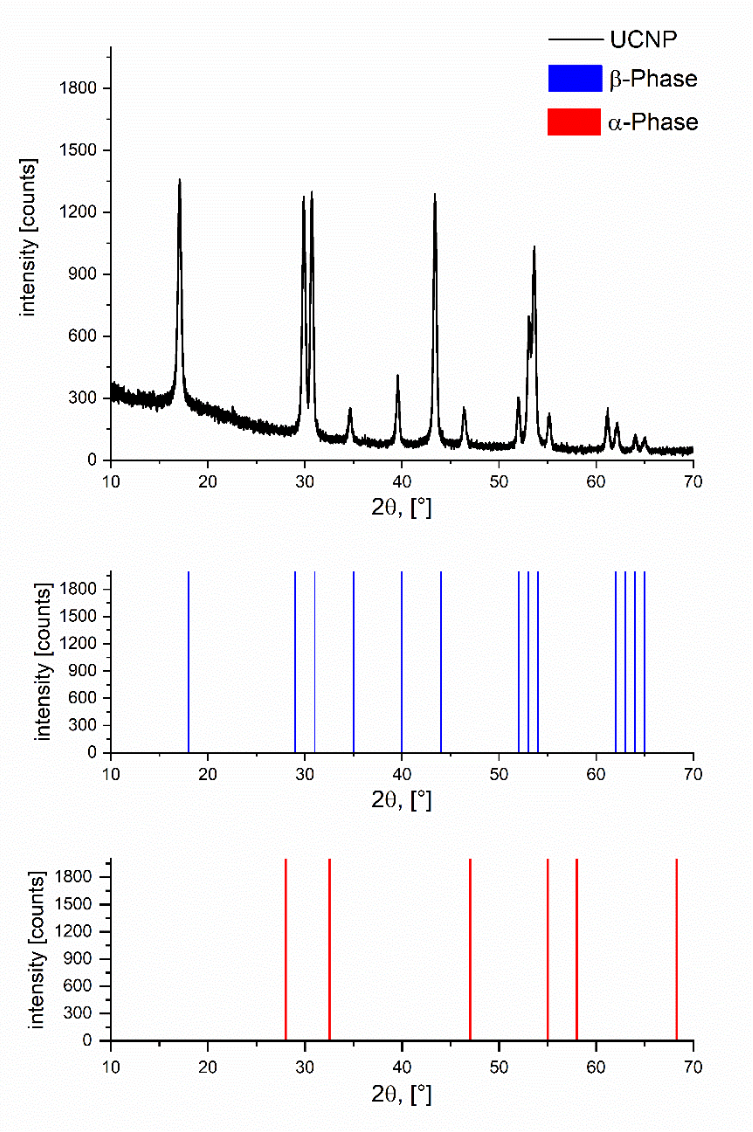

Figure 3: XRD diffractogram of the oleate stabilized upconversion nanoparticles, black spectrum. The blue bars refer to the reference values of the hexagonal β-phase crystal structure (JCPDS No. 28-1192), and the red bars refer to the reference values of the cubic α-phase crystal structure (JCPDS No. 77-2042) crystal structure of undoped $NaYF_4$.

Table S3: Average surface zeta potential of the upconversion nanoparticles of the different surface functionalizations

Sample ID	Zeta potential (mV)	PdI	Schematic representation
UC-PAA-20	-22.1 ± 0.9	0.36 ± 0.01	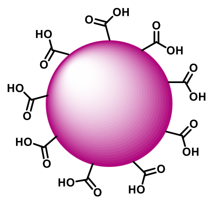
UC-PAA-30	-20.0 ± 0.46	0.24 ± 0.04	
UC-bare-20	32.8 ± 0.11	0.34 ± 0.01	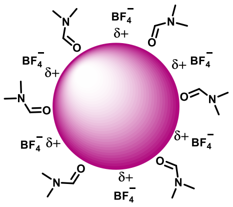
UC-bare-30	35.8 ± 0.55	0.40 ± 0.02	
UC-citrate-20	-18.9 ± 1.96	0.33 ± 0.04	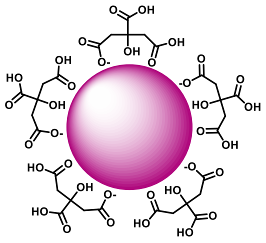
UC-citrate-30	-14.5 ± 2.03	0.79 ± 0.18	

The sensitizer, Yb^{3+} has a maximum absorption peak at 976 nm (Figure S3, A). Upon 976 nm excitation, $NaYF_4$: 20%Yb^{3+}, 2%Er^{3+} has three main emission peaks, two peaks in the green region of the visible spectrum (525 and 541 nm) and one peak in the red region (655 nm; Figure S3, B).

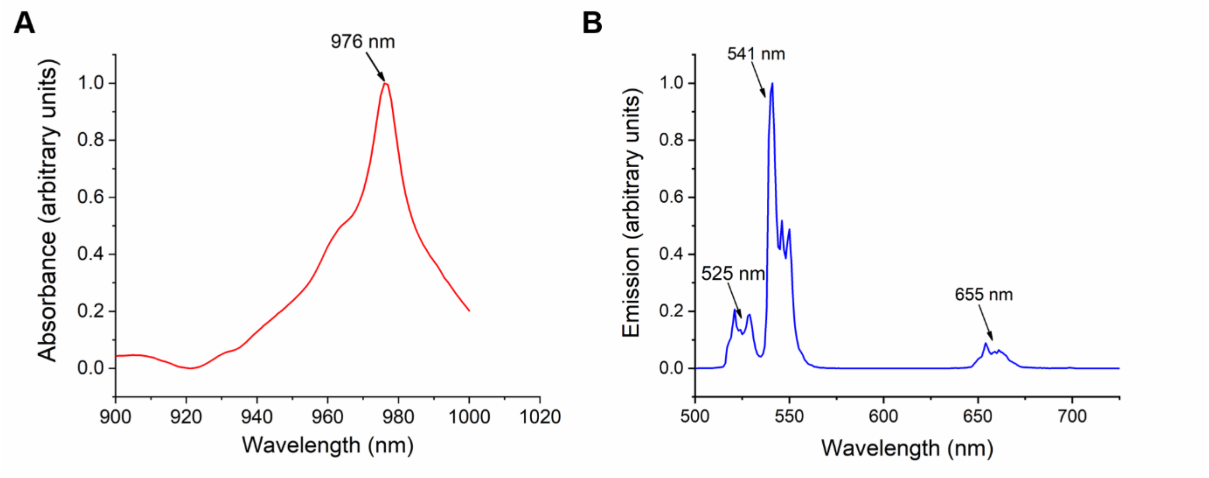

Figure S4: **A)** Absorbance of the upconversion nanoparticles showing an absorption maximum at 976 nm. **B)** UCL spectrum of the UCNP upon excitation at 976 nm revealing the green and red Er^{3+} emission bands.

5. Analytical Measurements for the Detection of the Released Ions

Table S4: Percentages of lanthanide ions released upon dissolution of UCNP in water. The concentration of the starting UCNP dispersion was 50 µg/L in water

Sample ID	% Y^{3+}	% Yb^{3+}	% Er^{3+}
UC-PAA-20	75.77	21.58	2.65
UC-PAA-30	75.60	21.58	2.82
UC-bare-20	75.25	21.58	3.17
UC-bare-30	79.51	17.36	3.12
UC-citrate-20	77.51	19.35	3.13
UC-citrate-30	75.36	21.64	2.99

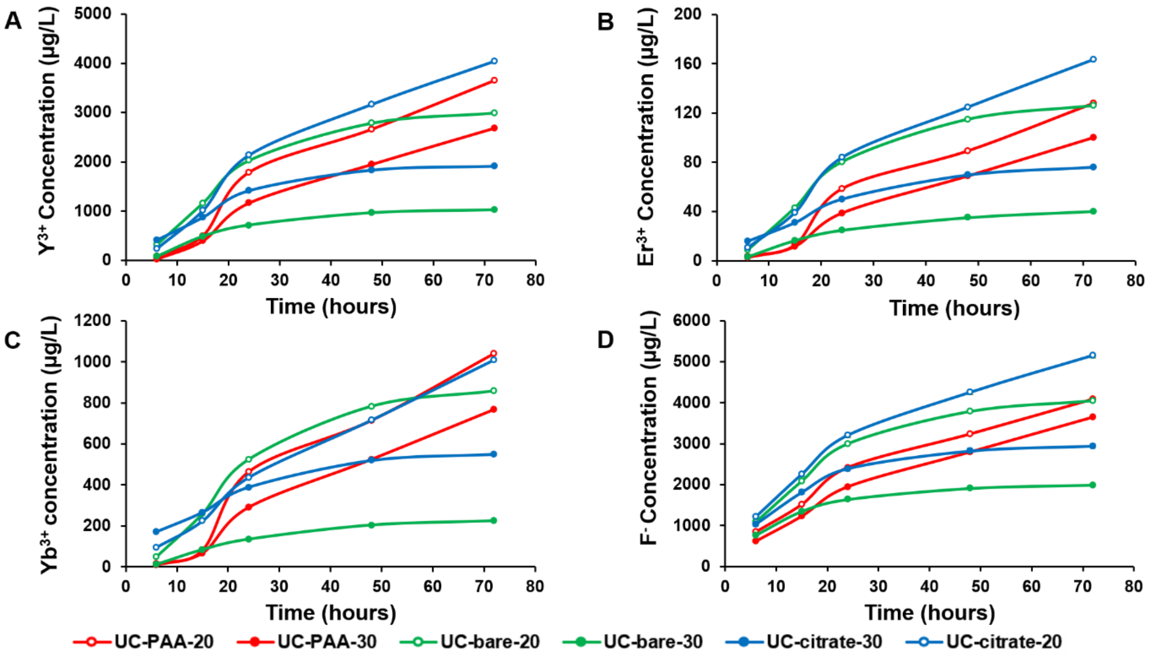

Figure S5: Concentrations of the ions released from upconversion nanoparticles of different particle sizes and surface functionalizations in water at room temperature. The starting concentration of the nanoparticles was 50 mg/L. Lanthanide ion concentrations were obtained from ICP/OES measurements of the supernatants after aging studies, while the fluoride ion concentrations were analyzed using a fluoride ion selective electrode. The solid lines are a guide to the eye.

Table S5: Results of the ISE measurements revealing the amount of fluoride ions released from the differently sized UCNP of varying surface chemistries upon aging in water for 72 hours. Two concentrations of UCNP in water were used, 5 and 50 mg/L.

Sample name	released F^- conc. [ppb]	Start conc.	X_F [mol%]
UC-PAA-20	1399	5 ppm	76.67
	4090	50 ppm	22.42
UC-PAA-30	1067	5 ppm	58.48
	3650	50 ppm	20.00
UC-bare-20	1576	5 ppm	86.37
	4050	50 ppm	22.20
UC-bare-30	1139	5 ppm	62.42
	1990	50 ppm	10.90
UC-citrate-20	1251	5 ppm	68.56
	5160	50 ppm	28.28
UC-citrate-30	1252	5 ppm	68.61
	2940	50 ppm	16.11

Table S6: Concentration and mole fraction of fluoride ions released upon aging of UCNP in water for 72 hours. The concentration of the starting UCNP dispersions were always 50 mg/L.

Sample ID	F^- (µg/L)	T	X_F (mol%)
UC-bare-20	4050	RT	22.20
	6640	37°C	36.39
UC-citrate-20	7210	RT	39.51
	7780	37°C	42.64
UC-SiO_2-thick	200	RT	1.096
	102	37°C	0.559
UC-SiO_2-thin	1830	RT	10.03
	4281	37°C	23.46
UC-mSiO_2-NH_3	2150	RT	11.78
	2694	37°C	14.76
UC-mSiO_2-NaOH	2990	RT	16.39
	3966	37°C	21.74

Table S7: Mole fraction of fluoride ions released upon UCNP aging in different aqueous environments. Starting concentration of UCNP: 50 mg/mL.

Sample ID	T	Aqueous medium	X_F [mol%]
UCNP-bare-20	RT	PBS	46.39
	RT	H_2O	22.20
	37 °C	DMEM	4.165
UCNP-citrate-20	RT	PBS	49.08
	RT	H_2O	39.51
	37 °C	DMEM	4.05
UCNP-SiO_2-thin	RT	PBS	19.03
	RT	H_2O	10.03
	37 °C	DMEM	1.261
UCNP-mSiO_2-NH_3	RT	PBS	17.45
	RT	H_2O	11.78
	37 °C	DMEM	1.096
UCNP-mSiO_2-NaOH	RT	PBS	21.66
	RT	H_2O	16.39
	37 °C	DMEM	1.315
UCNP-SiO_2-thick	RT	PBS	2.263
	RT	H_2O	1.096
	37 °C	DMEM	2.192

6. Luminescence Lifetime Measurements

Table S8: Yb^{3+} and Er^{3+} and luminescence lifetimes of UCNP-citrate-20 and UCNP-SiO_2-thin samples. Both samples were aged in water for different time intervals. The UCNP were then collected and redispersed in water for the lifetime measurements. Lifetime measurements were carried out in MilliQ water with a spectrofluorometer (Edinburgh Instruments, FLS-980) equipped with an electrically pulsed, 8 W 976 nm laser diode. The lifetimes of the Yb^{3+} and Er^{3+} emission bands were obtained from the decay profiles recorded at 1010 nm and 545 nm using tail fitting with a biexponential decay function.

Sample name	Aging time [hours]	Lifetime Er UC [µs]	Lifetime Yb DC [µs]
UCNP-citrate-20	0 h	66	47
UCNP-citrate-20	6 h	62	47
UCNP-citrate-20	24 h	63	41
UCNP-citrate-20	48 h	60	33
UCNP-SiO_2-thin	0 h	94	87
UCNP-SiO_2-thin	6 h	73	66
UCNP-SiO_2-thin	24 h	71	60
UCNP-SiO_2-thin	48 h	72	57

Table S9: Er^{3+} and Yb^{3+} luminescence lifetimes of sample UCNP-bare-20 aged in DMEM for different time intervals. The nanoparticles were collected and redispersed in water for measurements. Lifetime measurements were carried out with a spectrofluorometer (Edinburgh Instruments, FLS-980) equipped with an electrically pulsed, 8 W, 976 nm laser diode. The lifetimes of the Yb^{3+} and Er^{3+} emission bands were obtained from the decay profiles recorded at 1010 nm and 545 nm using tail fitting with a biexponential decay function.

Sample name	Aging time [hours]	Solvent	Lifetime Er UC [µs]	Lifetime Yb DC [µs]
UCNP-bare-20	0 h	H_2O	87	65
UCNP-bare-20	0 h	DMEM	87	77
UCNP-bare-20	12 h	DMEM	86	81
UCNP-bare-20	24 h	DMEM	85	81
UCNP-bare-20	48 h	DMEM	85	85

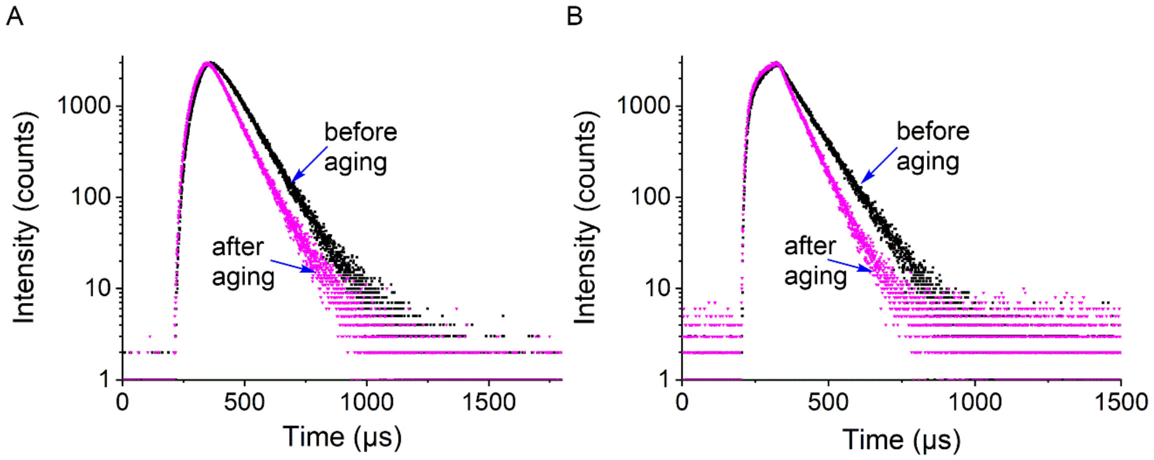

Figure S6: **A)** Er^{3+} and **B)** Yb^{3+} luminescence decay curves of unaged (magenta) and aged (black) UCNP-SiO_2-thin sample. UCNP were aged for 72 hours in water, then collected by centrifugation, and redispersed in MilliQ water for the measurements. Excitation wavelength: 976 nm. Yb^{3+} and Er^{3+} decays were detected at 1010 nm and 545 nm.

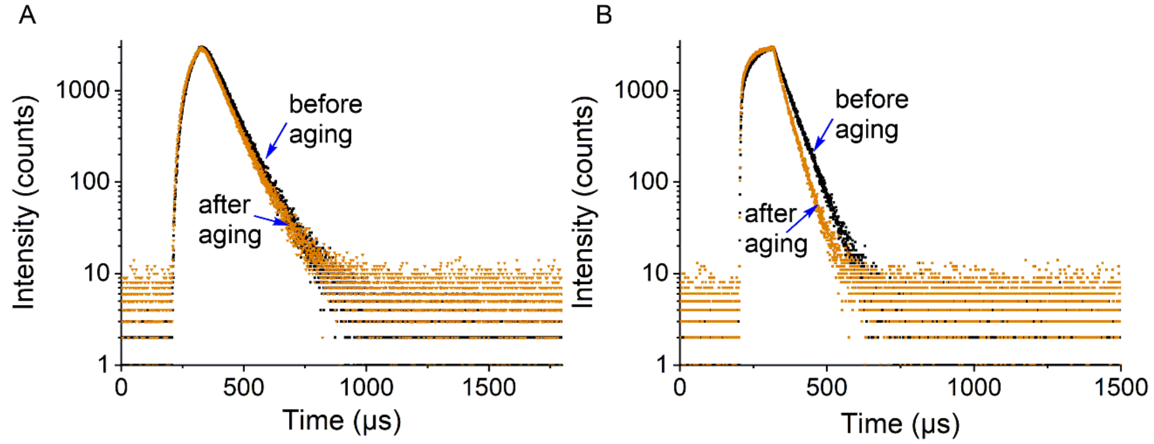

Figure S7: A) Er^{3+} and B) Yb^{3+} luminescence decay curves of aged (black curves) and unaged (orange curves) sample UCNP-citrate-20. Nanoparticles were aged for 72 hours in water, collected after aging, and redispersed in water for the measurements. Excitation wavelength: 976 nm, Yb^{3+} and Er^{3+} decays were detected at 1010 nm and 545 nm.

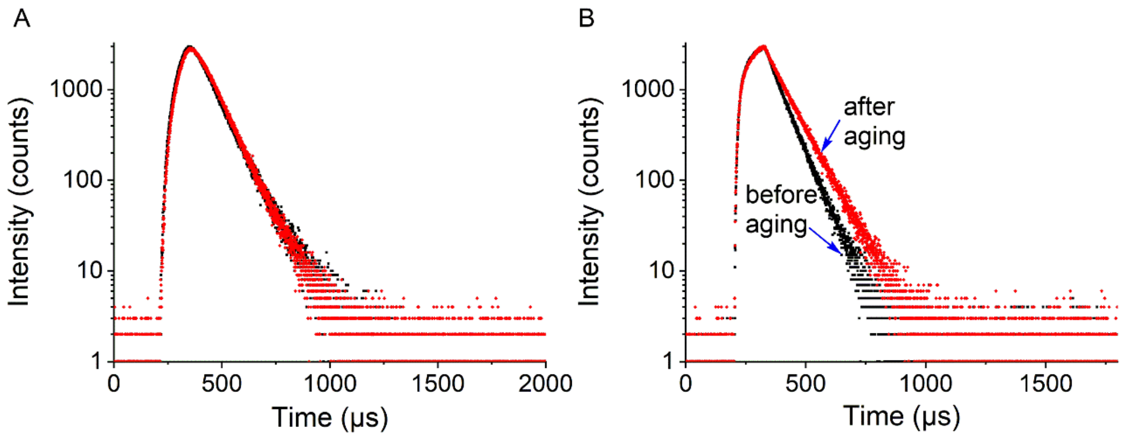

Figure S8: **A)** Er^{3+} and **B)** Yb^{3+} luminescence lifetime curves of aged (black curves) and unaged (orange curves) sample UCNP-citrate-20. Nanoparticles were aged for 72 hours in DMEM, collected after aging, and redispersed in water for the measurements. Excitation wavelength: 976 nm, Yb^{3+} and Er^{3+} decays were detected at 1010 nm and 545 nm.

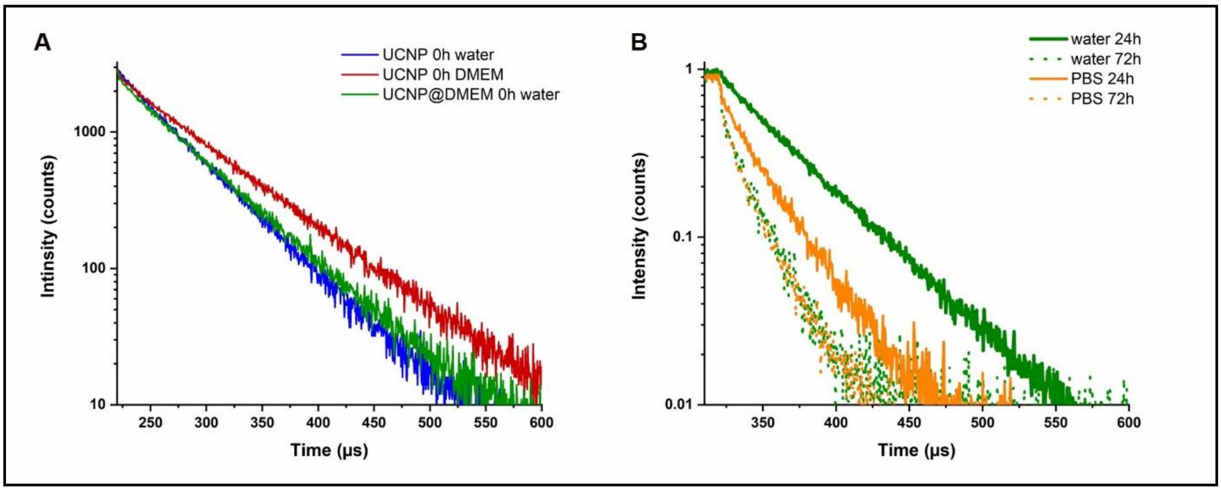

Figure S9: **A)** Normalized decay curves of the DC emission of Yb^{3+} of UCNP in water (blue), UCNP after immediate transfer to DMEM (UCNP@DMEM, red), and UCNP@DMEM after immediate transfer to water (green curve). **B)** Normalized decay curves of the Yb^{3+} DC emission of UCNP@DMEM aged in water for 24 hours (green line) and 72 hours (green dotted line) and in PBS for 24 hours (orange line) and 72 hours (orange dotted line). Excitation wavelength: 976 nm, Yb^{3+} and Er^{3+} decays were detected at 1010 nm and 545 nm.

7. X-ray Photoelectron Spectroscopic Measurements Results

Table 10: XPS results of unaged UC-bare-20 showing the elemental composition of the surface of the UCNP and the types of chemical bonding as suggested by the binding energies

Peak name	E_B/eV	Area/cps.eV	Sens. Fact.	Norm. Area	Quant./at.%	
Y 3d	159	313473.3	106180	2.952271	17	
C1s	285	29942.26	18783	1.594038	9.18	
O1s	524	15905	57121	0.2784399	1.6	
F1s	684	783160	81590	9.598722	55.27	
Na 1s	1071	250385.9	85092	2.942518	16.94	
Peak name	Peak hight/ Counts	Lorentzian	Position/eV	FWHM/eV	Abs. Area/ Counts eV	Rel. Area/ %
C-C, C-H	8229.1	0.1	284.921	1.4	12712	78.31
C-OR	1393.5	0.1	286.3808	1.4	2153	13.26
C=O	831.842	0.1	288.5549	1.4	1282	7.89
O-C=O	57.215	0.1	289.6822	1.4	87.72	0.54

Table 11: XPS results of UC-bare-20 after incubation in DMEM showing the elemental composition of the surface of the UCNP and the types of chemical bonding as suggested by the binding energies

Peak name	E_B/eV	Area/cps.eV	Sens. Fact.	Norm. Area	Quant./at.%	
Si 2p	99	66576.48	14080	4.728426	33.88	
C2p	200	17209.21	41363	0.4160509	2.98	
C1s	285	71412.04	18760	3.802558	27.25	
Ca1s	348	10017.33	972277	0.1029765	0.74	
N 1s	401	10884.16	34960	0.3113233	2.23	
O 1s	532	230073.1	57091	4.0299	28.88	
Na 1s	1072	48052.79	85062	0.5649132	4.05	
Peak name	Peak hight/ Counts	Lorentzian	Position/eV	FWHM/eV	Abs. Area/ Counts eV	Rel. Area/ %
C-C, C-H	13032.8	0.1	284.8346	1.4	20176	51.02
C-OR	6644.5	0.1	286.3934	1.4	10298	26.04
C=O	2271.6	0.1	288.2208	1.4	3523	8.91
O-C=O	1604.4	0.1	289.8536	1.4	2488	6.29
K 2p3	1345.4	0.1	293.2611	1.4	2083	5.27
K 2p1	633.882	0.1	295.9938	1.4	976.26	2.47
Peak name	Peak hight/ Counts	Lorentzian	Position/eV	FWHM/eV	Abs. Area/ Counts eV	Rel. Area/ %
-NR2	2091.6	0.2	399.4478	1.7	4061	73.65
-N-C=O	748.075	0.2	401.1823	1.7	1453	26.35

8. References

1 Li, Z., Zhang, Y. & Jiang, S. Multicolor Core/Shell-Structured Upconversion Fluorescent Nanoparticles. *Advanced Materials* **20**, 4765-4769, doi:10.1002/adma.200801056 (2008).

2 Wang, F., Banerjee, D., Liu, Y., Chen, X. & Liu, X. Upconversion nanoparticles in biological labeling, imaging, and therapy. *Analyst* **135**, 1839-1854, doi:10.1039/c0an00144a (2010).

3 Wang, F. *et al.* Simultaneous phase and size control of upconversion nanocrystals through lanthanide doping. *Nature* **463**, 1061-1065, doi:10.1038/nature08777 (2010).

4 Radunz, S. *et al.* Evolution of Size and Optical Properties of Upconverting Nanoparticles during High-Temperature Synthesis. *The Journal of Physical Chemistry C* **122**, 28958-28967, doi:10.1021/acs.jpcc.8b09819 (2018).

5 Wilhelm, S. *et al.* Water dispersible upconverting nanoparticles: effects of surface modification on their luminescence and colloidal stability. *Nanoscale* **7**, 1403-1410, doi:10.1039/c4nr05954a (2015).

6 Li, C., Liu, J., Alonso, S., Li, F. & Zhang, Y. Upconversion nanoparticles for sensitive and in-depth detection of Cu2+ ions. *Nanoscale* **4**, 6065-6071, doi:10.1039/c2nr31570j (2012).

7 Kembuan, C., Saleh, M., Rühle, B., Resch-Genger, U. & Graf, C. Coating of upconversion nanoparticles with silica nanoshells of 5–250 nm thickness. *Beilstein Journal of Nanotechnology* **10**, 2410–2421, doi:doi:10.3762/bjnano.10.231 (2019).

8 Stöber, W., Fink, A. & Bohn, E. Controlled growth of monodisperse silica spheres in the micron size range. *Journal of Colloid and Interface Science* **26**, 62–69, doi:10.1016/0021-9797(68)90272-5 (1968).

9 Nozawa, K. *et al.* Smart control of monodisperse Stöber silica particles: effect of reactant addition rate on growth process. *Langmuir : the ACS journal of surfaces and colloids* **21**, 1516–1523, doi:10.1021/la048569r (2005).

10 Dong, A. *et al.* A generalized ligand-exchange strategy enabling sequential surface functionalization of colloidal nanocrystals. *J Am Chem Soc* **133**, 998-1006, doi:10.1021/ja108948z (2011).

11 Carron, S. *et al.* Assembly of near infra-red emitting upconverting nanoparticles and multiple Gd(III)-chelates as a potential bimodal contrast agent for MRI and optical imaging. *Dalton Trans* **44**, 11331-11339, doi:10.1039/c5dt00919g (2015).

12 Juan, J., Cheng, L., Shi, M., Liu, Z. & Mao, X. Poly-(allylamine hydrochloride)-coated but not poly(acrylic acid)-coated upconversion nanoparticles induce autophagy and apoptosis in human blood cancer cells. *Journal of Materials Chemistry B* **3**, 5769-5776, doi:10.1039/c5tb00646e (2015).
